# Cross-platform analysis of global microRNA expression technologies

**DOI:** 10.1186/1471-2164-11-330

**Published:** 2010-05-26

**Authors:** Carole L Yauk, Andrea Rowan-Carroll, John DH Stead, Andrew Williams

**Affiliations:** 1 Environmental Health Sciences and Research Bureau, Health Canada, Ottawa, ON K1A 0K9, Canada; 2 Institute of Neuroscience, Carleton University, Ottawa, ON K1S 5B6, Canada

## Abstract

**Background:**

Although analysis of microRNAs (miRNAs) by DNA microarrays is gaining in popularity, these new technologies have not been adequately validated. We examined within and between platform reproducibility of four miRNA array technologies alongside TaqMan PCR arrays.

**Results:**

Two distinct pools of reference materials were selected in order to maximize differences in miRNA content. Filtering for miRNA that yielded signal above background revealed 54 miRNA probes (matched by sequence) across all platforms. Using this probeset as well as all probes that were present on an individual platform, within-platform analyses revealed Spearman correlations of >0.9 for most platforms. Comparing between platforms, rank analysis of the log ratios of the two reference pools also revealed high correlation (range 0.663-0.949). Spearman rank correlation and concordance correlation coefficients for miRNA arrays against TaqMan qRT-PCR arrays were similar for all of the technologies. Platform performances were similar to those of previous cross-platform exercises on mRNA and miRNA microarray technologies.

**Conclusions:**

These data indicate that miRNA microarray platforms generated highly reproducible data and can be recommended for the study of changes in miRNA expression.

## Background

DNA microarray technologies are powerful tools that can be used to quantify differences in the amount of specific DNA or RNA sequences between samples. Recently, application of this technology has extended to the measurement of changes in the abundance of microRNAs (miRNA). MiRNAs are a class of small non-coding RNAs (typically 21-25 nucleotides in length) that bind to mRNAs to regulate protein expression either by blocking translation or by promoting degradation of the mRNA transcript [1]. These molecules are implicated in a large number of biological processes and human diseases (e.g., [2-8]). As such, hundreds of papers in the past five years have been published using microarray technologies to explore changes in miRNA abundance.

Despite its widespread application, only three papers have systematically investigated the reproducibility of results produced between different miRNA detection technologies [9-11], with only one specifically comparing microarray platforms [11]. Early studies examining the reproducibility and correlation among microarray technologies for the detection of mRNA gene expression revealed very poor correlations, in part related to sub-optimal protocols, incomplete or incorrect annotation of early platforms, incorrect probe-matching between technologies, differences in data normalization, inadequate pre-filtering of probes that had low signal intensities, and lab-lab variation in technical abilities (reviewed in [12]). Later large-scale studies on more accurately annotated platforms applied appropriate probe-matching techniques, mathematical modeling and filtering techniques to reveal a very high correlation among mRNA microarray detection technologies both within and across laboratories (e.g., [13-16]). It is expected that the lessons learned from these early mRNA platforms will result in fewer problems associated with reproducibility among novel array-based nucleic acid technologies in the future. Nevertheless, as miRNA technologies are new and significant differences exist in the probe design and experimental protocols associated with the various commercially-available platforms, it is essential to investigate the correlation and reproducibility among these technologies. Various sources of technical and analytical variability are associated with miRNA profiling and it has been suggested that much more work is required to identify and minimize these technical variables and increase reliability and credibility of miRNA profiles (reviewed in [17]).

In the present study we investigate intra- and inter-platform correlation among 4 miRNA microarray technologies (Agilent Technologies, Exiqon, Invitrogen, LC Sciences) using commercially-available reference materials. We also investigate the rank correlation of miRNA expression on microarray platforms and miRNA expression using TaqMan qPCR-arrays.

## Results

We created two reference samples using commercially-available mouse reference RNA. The two references were selected to ensure differences in miRNA abundance between the samples (i.e., to optimize the number of differentially expressed miRNA), similar to the methods described by Irizarry *et al*. [18]. Reference 1 was created by pooling RNA derived from mouse testicle, ovary and from embryos, while liver, heart and lung were pooled to create Reference 2. Aliquots from these four references were hybridized to four genome-wide microarray technologies (Agilent, Exiqon, Invitrogen NCode and LC Sciences). The results from these arrays were compared to each other, and were then compared to the TaqMan miRNA PCR arrays produced from the same samples. The Agilent and LC Sciences arrays were carried out in 1 color, with 2 replicates per Reference pool. The Exiqon and Invitrogen NCode arrays were analyzed in two colors. For the two-color arrays, Reference 1 was labelled with Cy3 (Exiqon) or Alexa Fluor 3 (Invitrogen NCode), with Reference 2 labelled with Cy5 or Alexa Fluor 5, and then a dye swap was carried out. This was done twice for a total of 4 microarrays per platform. All microarray analyses presented herein were based on a sample size of 4 (two replicates of Reference 1 and two replicates of Reference 2 for each platform). To ensure that the different platforms were maximally comparable, each of the two-color platforms were also analysed in one-color format. The Exiqon platform was thus analysed for the Cy3 channel only, with the NCode platform analysed for the Alexa Fluor 5 channel only. In general, inter-platform correlations were highest for both platforms when data were analyzed in two-colors, though this reduced intra-platform reliability specifically for the NCode platform.

One process that can introduce discrepancies between the results produced by different platforms is the selection of the appropriate normalization algorithm. Various methods have been proposed for miRNA arrays. In the present study, microarray data from each of the technologies were normalized using a cyclic lowess approach for 1-color analyses, and a dye swap with lowess normalization for 2-colors. Initial analysis revealed that the platform correlations were slightly higher using this approach than using a quantile normalization (data not shown). Cyclic lowess normalization assumes an equal proportion of up-regulated and down-regulated probes, and this assumption was not violated in the current dataset (data not shown).

MiRNA sequences are extremely short, and thus the problems associated with probe matching that complicated the comparison of gene expression profiles across technologies are less relevant. However, the various platforms' probes were developed using different miRBase databases and thus should not be matched by name alone. Thus, to compare across platforms probes were matched by sequence. Technical replicates of probes were spotted on all of the array technologies; the medians of these technical replicates were used for the subsequent analyses. Using this approach there were 189 probes in common across the 4 microarray technologies. Among these probes, 54 yielded signal intensities that were significantly above background (i.e., 'present') on all platforms and were also included on the TaqMan arrays. Venn diagrams for present probes are found in Additional Files 1 and 2 (File 1: Figure S1; File 2: Figure S2). In general the detection of probes between pairwise comparisons was quite similar, with no single platform greatly influencing the overall number of probes in common. When comparing both within and between platforms, correlation analyses were performed on two different probe sets. First, to ensure calculations were based on maximum numbers of miRNAs, correlations were determined for all miRNAs that were 'present' on both platforms being compared. Second, in order to ensure a fair comparison across all platforms, correlation analyses were performed on the subset of 54 miRNAs present on all platforms.

We examined within-platform quality metrics and reproducibility to get a general sense of the performance of each platform. The numbers of miRNAs detected within the platforms were very similar (122 miRNAs present for Agilent, 131 for Exiqon, 118 for Invitrogen NCode, and 131 for LC Sciences). A rank correlation analysis was carried out to evaluate the precision of the platforms (i.e., correlation of technical replicates). Spearman correlation analysis revealed a very high level of reproducibility within platforms for all four of the miRNA microarray platforms (Table 1). The poorest reproducibility was observed for the Invitrogen NCode platform, particularly when two-color data were analyzed. Closer inspection revealed that signal quality was relatively low for the Alexa Fluor 3 channel which led to greater differences in comparisons between the dyes. Exclusion of data from this channel through single-color data analysis greatly improved reproducibility to 0.850. Reliability of this platform may have also been compromised by printing arrays in-house, and indeed, correlations of the other commercial platforms were higher (0.913-0.995). We note that a very high level of within-platform correlation was obtained for LC Sciences, despite using sub-optimal hybridization conditions (starting total RNA was limiting).

**Table 1:** Within platform correlations for miRNA arrays.

	Probe Technology	MiRBase version	One or two-color analysis	Number of miRNAs considered	Correlation	Standard Error	95% Confidence intervals
Agilent Mouse miRNA (P/NG4472A)	60 mer probes with hairpin loop structure and 5'G residue complement to the C residue added to the microRNA during labelling	MirBase 12	One	122	0.995	0.0014	0.991-0.997
				54	0.995	0.0035	0.984-0.997
LC Science Mouse miRNA Microarray MRA-1002	μParafloTM Technology uses proprietary in-situ oligo synthesis, microfluidic reaction cells, and novel photochemistry coupled with photolithography; full content flexibility	MirBase 12	One	131	0.978	0.0068	0.961-0.987
				54	0.981	0.0105	0.952-0.992
Exiqon miRCURY LNA™ microRNA Array kit	LNA Locked Nucleic Acids are conformationally locked into a favourable confirmation for miRNA binding	MirBase 11	Two	131	0.914	0.0189	0.870-0.944
				54	0.913	0.0258	0.848-0.948
			One	125	0.874	0.0330	0.798-0.925
				54	0.802	0.0723	0.631-0.910
Invitrogen NCode Multi-Species miRNA Microarray Probe Set V2	34-44 nucleotide probes designed as dimers of sequences complementary to mature miRNAs, trimmed to reduce melt temperature variability between probes.	MirBase 9	Two	118	0.706	0.0544	0.586-0.798
				54	0.816	0.0532	0.686-0.892
			One	124	0.853	0.0338	0.775-0.907
				54	0.850	0.0553	0.716-0.928

Spearman correlations and the 95% confidence intervals are presented for the miRNAs that were present within a platform from the sequence-matched list, as well as the 54 miRNAs that gave detectable signals on all four platforms.

A rank correlation analysis was applied to compare across the technologies, where the two reference samples were used to derive log ratios (i.e., relative expression). Log ratios were generated using all combinations of references pairs. A Spearman correlation was then used to examine the relationship across technologies (Table 2; see Additional file 3: Table S1 for complete dataset). For the two-color platforms, these comparisons were based on both one- and two-color analyses, each of which are presented in Table 2. However, discussion of the data comparing Exiqon and Invitrogen NCode platforms to other arrays will be restricted to the two-color analyses, given that these platforms were designed as two-color platforms and were run in two color form, and therefore there is potential for cross-talk or competitive hybridization between the channels. A high level of correlation was observed between all array platforms. Correlation coefficients ranged from 0.765-0.949 in analyses based on miRNAs present on the two platforms being compared, or 0.731-0.949 based on the 54 miRNAs present across all platforms. Analysis of the 54 miRNAs in common revealed a higher correlation coefficient for Agilent vs Exiqon compared to the other platform contrasts. However, there was a high degree of overlap of the confidence intervals across the correlation analyses. Scatterplots were generated for present and absent miRNAs (see Additional file 4). Although the present probes revealed higher R^2 ^values (range 0.431-0.867), probes within the background were also positively linearly associated (R^2 ^range 0.214-0.741).

**Table 2:** Between platform pairwise correlations for miRNA arrays.

	Agilent	LC Sciences	Exiqon 2	Exiqon 1	NCode 2	NCode 1
Agilent		0.794 0.676-0.870 (71)	0.949 0.905-0.970 (72)	0.892 0.811-0.940 (69)	0.770 0.628-0.863 (70)	0.768 0.623-0.865 (72)
LC Sciences	0.757 0.617-0.849 (54)		0.824 0.730-0.882 (72)	0.766 0.646-0.843 (70)	0.765 0.649-0.843 (71)	0.663 0.491-0.790 (75)
Exiqon 2	0.949 0.892-0.974 (54)	0.794 0.664-0.876 (54)		0.943 0.895-0.967 (81)	0.808 0.680-0.890 (71)	0.750 0.604-0.849 (74)
Exiqon 1	0.867 0.756-0.931 (54)	0.722 0.559-0.832 (54)	0.927 0.849-0.966 (54)		0.823 0.702-0.897 (68)	0.771 0.631-0.864 (71)
NCode 2	0.843 0.715-0.918 (54)	0.731 0.562-0.843 (54)	0.869 0.751-0.934 (54)	0.884 0.778-0.943 (54)		0.891 0.800-0.943 (76)
NCode 1	0.822 0.664-0.917 (54)	0.672 0.446-0.830 (54)	0.814 0.664-0.905 (54)	0.822 0.676-0.909 (54)	0.935 0.865-0.966 (54)	

Spearman correlations and the 95% confidence intervals are presented for miRNA present in individual pairwise-contrasts (above diagonal) or using the 54 sequence-matched miRNAs with detectable signals on all four miRNA platforms. Numbers in brackets indicate the number of miRNA probes used in deriving the analysis.

Because qRT-PCR is used to validate the results of microarray experiments, we used TaqMan miRNA arrays to explore the correlation of arrays with qRT-PCR. This analysis is aimed at evaluating the potential accuracy of the microarrays, as described in [18]. The differences on the log_2 _scale were multiplied by -1 in order to derive a positive correlation coefficient, since low Ct values are associated with high expression (i.e., the opposite of DNA microarrays). All miRNAs that were present on the miRNA arrays yielded Ct values equal to, or less than 25. Thus, these miRNAs exhibited Cts that were well within the range of detection of this method [19]. Spearman correlation analysis revealed a high level of agreement between the TaqMan and miRNA arrays (Table 3). The average correlation coefficients were 0.68 (miRNA present in a pairwise contrast) and 0.65 (using the 54 miRNAs present across all platforms). Irrespective of the analysis employed (one- or two-color, or miRNAs present in each pair-wise contrast, or all miRNAs present in all platforms), correlations ranged from 0.642-0.775. The only exception was in correlations between LC science arrays and RT-PCR using the restricted set of 54 miRNAs, which gave a correlation of 0.51. This correlation was significantly lower than those observed with some of the other platforms, though significance was marginal and is lost after family-wise error rate correction (Additional File 5: Table S2). Concordance correlation coefficients (CCCs) were calculated for the 54 miRNAs in common and present, as well as for the miRNAs that were present on an individual microarray platform. These data are summarized in Table 4. In contrast to the rank correlation analysis, LC Sciences exhibited the highest CCC in this analysis, with NCode 2 colour demonstrating the lowest. However, analysis of the 54 miRNA in common among all platforms revealed a high overlap among the 95% confidence intervals, and thus did not reveal any significant differences between the platforms.

**Table 3:** MiRNA array correlations with TaqMan arrays.

	One or two-color analysis	Number of miRNAs considered	Correlation	Standard Error	95% Confidence intervals
Agilent	One	122	0.692	0.0633	0.553-0.799
		54	0.653	0.1073	0.414-0.831
LC Science	One	131	0.680	0.0590	0.548-0.782
		54	0.506	0.1215	0.241-0.762
Exiqon	Two	131	0.700	0.0569	0.575-0.799
		54	0.656	0.1079	0.412-0.835
	One	125	0.700	0.0598	0.567-0.804
		54	0.691	0.1009	0.467-0.855
Invitrogen NCode	Two	118	0.642	0.0567	0.517-0.737
		54	0.775	0.0850	0.575-0.907
	One	124	0.659	0.0573	0.533-0.759
		54	0.730	0.0896	0.521-0.873

Spearman correlations and the 95% confidence intervals are presented for all the miRNAs that were present on a platform, or using the 54 sequence-matched miRNAs that were detected by all four microarray technologies.

**Table 4:** Correlation between miRNA arrays and TaqMan arrays.

	One or two-color analysis	Number of miRNAs considered	CCC	95% Confidence intervals
Agilent	One	122	0.416	0.337-0.488
		54	0.405	0.268-0.527
LC Science	One	131	0.545	0.454-0.624
		54	0.610	0.451-0.732
Exiqon	Two	131	0.461	0.371-0.542
		54	0.532	0.377-0.657
	One	125	0.409	0.329-0.483
		54	0.459	0.320-0.579
Invitrogen NCode	Two	118	0.314	0.241-0.383
		54	0.435	0.315-0.540
	One	124	0.225	0.225-0.281
		54	0.344	0.243-437

Concordance correlation coefficients and the 95% confidence intervals are presented for all the miRNAs that were present on a platform, or using the 54 sequence-matched miRNAs that were detected by all four microarray technologies.

## Discussion

New technologies for the analysis of changes in miRNA expression are widely used but have not been adequately validated. We examined within and between platform correlation of four DNA array technologies for miRNA analysis as well as TaqMan PCR arrays. Within-platform correlations provide an estimate of the array reproducibility, while comparison to RT-PCR provides insight into the accuracy of the platforms as described in Irizarry *et al*. [18]. Two distinct pools of commercially-available reference materials were selected in order to maximize differences in miRNA content. All analyses were based on the log ratios of these two distinct samples as recommended in prior cross-platform microarray publications [14,18,20,21]. Aliquots from the same reference samples were used for all technologies to ensure comparability. Because LC Sciences was the last technology that we explored, miRNA concentration was limiting for this platform. As such, the protocol used was not according to the supplier's recommendation. Thus, we expect the results of this platform would improve if more total RNA was used. Probes across microarray technologies were matched by sequence to avoid errors in annotation resulting from the development of probes from different versions of miRBase. Filters were applied to remove miRNAs that gave signal intensities within the background, and the same normalization protocol was applied across the technologies. All analyses were carried out on a subset of genes that passed all these quality metrics. Moreover, two color platforms were also examined using a one-color approach to minimize differences across technologies.

Using this stringent data handling protocol we calculated the within-platform variability to determine the precision of the platforms [18]. This analysis revealed a high level of reproducibility within a given technology (Table 1). The Invitrogen NCode array exhibited the lowest within-platform correlation. This may be expected as these arrays were printed in-house, and both quality and reproducibility of a commercial product should be much higher. Moreover, we obtained poor signal quality on one of the channels, and a one-color analysis of this platform greatly improved reliability. Indeed, with the exception of the two-color NCode analysis, within-platform analyses revealed very high Spearman correlation for all pairwise contrasts, ranging from 0.850-0.995. These within platform correlation coefficients are within the same range as those of previous cross-platform and cross-institute exercises on mRNA microarray platforms (e.g., [18,21]) and microRNA technologies [11]. For example, Sato *et al*. obtained correlation coefficients for miRNA arrays that ranged from 0.83 to 0.99, depending on the tissue origin of the miRNA samples [11]. These data indicate that the four microarray platforms generated highly reproducible data that are in-line with previous cross-platform papers.

Further analyses also revealed a high level of reproducibility across technologies. Spearman correlation coefficients were generated for each possible pairwise contrast for each of the microarray technologies using miRNAs that were present across all platforms (n = 54) or in common between specific pairwise contrasts (Table 1). Scatterplots were produced for both present and absent miRNAs (Additional file 4). Spearman analysis compares the log ratio ranks (Reference 1 to Reference 2) to determine whether the distribution of fold changes is similar. Focusing on the two-color analyses for Invitrogen NCode and Exiqon (given that this is how these platforms were designed and hybridized), the average cross-platform correlation coefficient for all platform comparisons was 0.82 (range of 0.731-0.949) using the 54 miRNAs present across all platforms, with a large amount of overlap in the confidence intervals. Scatterplots for all pair-wise comparisons (Additional file 4) yielded very similar findings. The cross-platform correlations are consistent with previous work on mRNA expression arrays [14,16] and with the single previous study examining miRNA array platforms [11]. For example, Spearman rank correlation coefficients for cross-platform analyses ranged from 0.590 to 0.941 in the MAQC consortium [21]. Rank correlation analysis of miRNA arrays revealed a median rank correlation of 0.55 (using only detectable miRNAs); the highest correlation found was 0.87 [11]. Thus, the results of the present study reveal correlation coefficients that overlap with the high end of these previous cross-platform analyses. As such, we conclude that results generated by these different technologies are highly concordant.

The highest correlation was found between Agilent and Exiqon (Table 2). Results for this comparison were significantly higher than most of the other comparisons. We routinely use the Agilent platform for all of our gene expression studies, and thus expected within-and between-platform correlations for Agilent to be high. Moreover, the Agilent and Exiqon platforms were the first experiments to be run, and were run inside our laboratory, while LC Sciences and NCode were done in other laboratories (see Methods). As LC Sciences was the last platform analyzed, the amount of RNA available was well below the company's recommended protocol. Thus, we caution that the finding that one platform is better than the others, or that Exiqon and Agilent produce more correlated data, may not generally extend beyond the present experiment. Indeed, our primary interpretation of the data is that the platforms show a high level of similarity to each other despite being hybridized in different labs, at different times, with different protocols and amounts of expertise.

We examined the correlation of the log ratios between the array platforms and miRNA expression measured using TaqMan arrays, as qRT-PCR is often considered to be the 'gold standard' for validation. All miRNA that were 'present' on the array technologies yielded Ct values that were equal to, or less than, 25. The correlation coefficients from this analysis ranged from 0.506-0.775. This is much higher than a previous publication examining the correlation between TaqMan PCR arrays and miRNA arrays, which yielded a correlation coefficient of 0.44, suggesting considerable variability between the approaches [10]. However, these numbers are in-line with the previous study on miRNA arrays that compared several platforms to TaqMan arrays, and found a range of correlation coefficients from 0.44 to 0.86 (including 0.85 for Agilent and 0.67 for Exiqon) [11]. This analysis is aimed at estimating the potential accuracies of the platforms. Using the 54 miRNAs that were present among all platforms, LC Sciences exhibited the lowest rank correlation coefficient with TaqMan, however, this may be due to small sample size. Indeed, the correlation coefficient for the 122 miRNAs present on that platform was 0.680, in-line with the other platforms. Moreover, analysis of CCCs for this platform (against TaqMan arrays) revealed the highest coefficients. CCC incorporates measures of both accuracy and precision in one index and is proposed to be a universal measure of study quality [22,23]. The precision is not based on ranks, and thus will give slightly different result than the above Spearman rank correlation analysis. The ranges of CCCs were in-line with data produced for Affymetrix gene expression data against RT-PCR [23]. In the present analysis, LC Sciences exhibited the highest CCC, however, comparisons among the 54 sequence-matched miRNAs that were present on all platforms revealed overlap among the confidence intervals (i.e., no significant differences among the platforms). These combined data indicate that there were minimal differences in correlation with TaqMan among the technologies, and all platforms perform well relative to mRNA expression arrays.

The present study is complementary to a recent elegant paper by Sah *et al*. [24], which employed a very different experimental design to evaluate performance of multiple miRNA microarray platforms. In our study, we compare different biological samples to evaluate reliability of arrays within and between platforms, as well as between technologies (i.e., microarray vs. RT-PCR). In contrast, Sah *et al*., used dilution series of spike-in positive controls on four microarray platforms to evaluate the platforms. Specifically, they determined accuracy/sensitivity (defined as whether arrays revealed expected changes in signal intensity following known changes in spike-in concentration, over a wide range of concentrations) and specificity/precision (changes in signal intensity specific only to probes for the manipulated spike-in controls, or changes observed for other miRNAs for which signal intensity is expected to be unaltered). As with our study, while differences were detected between the platforms, all four arrays performed well in each measure of array quality.

## Conclusions

We conclude that the knowledge acquired from early work on the technical issues related the protocols and data processing methods applied to gene expression microarrays have greatly facilitated the rapid implementation of highly-reproducible data using miRNA arrays. The four platforms examined in the present study show high levels of both within- and between-platform reproducibility. The platforms also correlate well with TaqMan arrays. We would recommend any of the methodologies listed in this paper for the study of changes in miRNA expression.

## Methods

### Reference miRNA materials

Reference RNA was developed from FirstChoice^® ^mouse Total RNA including the small fraction (Catalogue # AM7800-AM7828, Ambion, Streetsville, ON). Two references were made from the various stocks. Reference # 1 was a pool of equal amounts of total RNA from mouse testicle, ovary and 10-12 day embryo. Reference #2 was a pool of equal parts total RNA from mouse liver, heart and lung. Aliquots from the reference RNA pools were analyzed in the following order: Agilent (in-house), Exiqon (in-house), NCode (outsourced to Carleton University), TaqMan PCR arrays (out-sourced to the University of Montreal, Montreal, Quebec), followed by LC Sciences (outsourced to LC Sciences in Houston, Texas) over the period of June-September in 2009.

### miRNA expression profiling using Agilent arrays

Agilent miRNA arrays were performed in one color at Health Canada, Ottawa, with all samples on a single 8_x_15k microarray, according to the manufacturer's instructions (Agilent Technologies). Briefly, 100 ng reference RNA was dephosphorylated by incubation with calf intestinal phosphatase at 37°C for 30 min and denatured using 100% DMSO at 100°C for 5 min. Samples were labelled with pCp-Cy3 using T4 ligase by incubation at 16°C for 1 hour. Each labelled RNA sample was hybridised to an individual array on 8_x_15K format Agilent mouse miRNA array slides, with each array containing probes for 567 mouse miRNAs and 10 mouse gamma herpes virus miRNAs. Four arrays were hybridised with Reference #1, and four arrays were hybridized with Reference #2. Hybridisations were performed in SureHyb chambers (Agilent) for 20 hours at 55°C. Arrays were washed according to manufacturer's instructions and scanned at a resolution of 5 μm using an Agilent G2505B scanner. Data were acquired using Agilent Feature Extraction software version 9.5.3.1. One array yielded a very poor hybridization signal and did not pass the Agilent quality control metrics in the Feature Extraction software. Two arrays from each Reference sample were randomly chosen for the subsequent analyses.

### miRNA expression profiling using Exiqon arrays

Exiqon arrays were performed in two colors at Health Canada, Ottawa, according to manufacturer instructions. One microgram of each mouse Reference RNA sample was labelled with a Hy5 fluorophore (Exiqon, Woburn, MA, USA) and co-hybridised with 1 μg of the other Reference RNA standard in Hy3. Two replicates were performed as well as two dye swaps (i.e., a total of 4 arrays hybridized). Labelling reactions were performed using Exiqon's miRCURY LNA miRNA power labelling kit, according to the manufacturer's protocol. Briefly, 5' phosphates were removed using calf intestinal phosphatase. Hy3 or Hy5 fluorescent molecules were attached enzymatically to the 3' end of the miRNAs. Hy3 and Hy5 labelled samples were co-hybridised to Exiqon multi-species miRCURY LNA miRNA array slides (version 11.0), containing ~1700 capture probes covering all human, mouse and rat miRNAs annotated in miRBase 11.0. Hybridisations were performed in SureHyb chambers (Agilent) for 16 hours at 56°C. Slides were washed according to manufacturer's instructions and scanned at 5 μm resolution using an Agilent G2505B scanner. Data were acquired using Agilent Feature Extraction software version 9.5.3.1, using an FE protocol available on-demand from Exiqon.

### miRNA expression profiling using Invitrogen NCode arrays

NCode arrays (Invitrogen) were performed in two colors at Carleton University, Ottawa, according to manufacturer instructions. Custom arrays were printed in house using the Invitrogen NCode Multi-Species miRNA Microarray Probe Set V2 (Invitrogen) which includes 1140 probes covering six different species, including 427 mouse miRNA probes designed from the Sanger miRBase Sequence Database, Release 9.0. The probe set was modified to incorporate dilution series for endogenous miRNAs and spike-in controls, print-tip carry-over controls, and random hexamer negative controls based on the design described by Yauk et al [25]. Following Invitrogen guidelines, probes were dissolved at 30 μM in Pronto!™ Epoxide Slide Spotting Solution (Corning) and printed on Epoxide-Coated Slides (Corning) using the VersArray ChipWriter Pro (BioRad), with each spot replicated 6 times per array. Arrays were pre-soaked and pre-hybridized according to manufacturer's instructions (Corning). Two replicates and two dye-swaps (i.e., 4 arrays) were performed. For each labelling reaction, 0.5 μg of total reference RNA was spiked with 40 fmoles NCode Microarray Positive Control (Invitrogen) and labelled using the NCode Rapid miRNA Labelling System according to manufacturer's instruction (Invitrogen). Briefly, polyA polymerase is used to add a poly(A) tail to the RNA, to which is then ligated a DNA polymer carrying ~15 molecules of the fluorophore (Alexa Fluor^® ^3 or 5). Ligation is mediated by an oligo(dT) bridge whose sequence matches both the poly(A) tail of the RNA, and the fluorophore-labelled DNA polymer. Arrays were hybridized 10 hours at 52°C. In a modification of the standard protocol, hybridizations were performed using Agilent hybridization chambers within a rotisserie oven, which required dilution of the hybridization mix to a total volume of 120 μl, by addition of BSA (8 mg/ml) and Enhanced Hybridization Buffer (Invitrogen). Arrays were washed and dried by centrifugation according to manufacturer's instructions. To protect dyes against ozone-mediated degradation, 0.1 mM dithiothreitol was added to each wash solution, and slides were dried and stored in sealed tubes containing 20 μl of ≥ 98% 2-mercaptoethanol, ensuring that there was no contact between the reducing agent and the array. Arrays were scanned with a GenePix 4000B laser scanner (Molecular Devices) with a scan resolution of 5 μm, and PTM between 620 to 660 V. Non-background-subtracted signal intensity was determined using Imagene 8.0 image analysis software (Biodiscovery, Inc.).

### miRNA expression profiling using LC Sciences

LC Sciences arrays were performed in one color by the company at the LC Sciences Headquarters (Houston, TX) using two replicates of each reference pool. This platform contains all known mouse miRNA from the Sanger miRBase database (release 14.0). The assay used lower starting quantities of total RNA because RNA was limiting (recommended 5-8 μg). Thus, the protocol was sub-optimal for this platform. Briefly, 1 μg of total reference RNA was extracted with Trizol reagent (Invitrogen) and size fractionated using a YM-100 Microcon filter (Millipore). Small RNAs less than 300 nucleotides in length were extended with a poly(A) tail using poly(A) polymerase. Poly(A) tails were ligated with an oligonucleotide tag for subsequent dye staining. Hybridizations were carried out overnight on μParaflo microfluidic chip using a micro-circulation pump (Atactic Technologies) [26,27] using 100 μL 6× SSPE buffer (0.90 M NaCl, 60 mM Na_2_HPO_4_, 6 mM EDTA, pH 6.8) containing 25% formamide at 34°C overnight. Each detection probe on the microfluidic chip consisted of a chemically-modified nucleotide coding segment complementary to target microRNA or other RNA (control sequences), and a spacer segment of polyethylene glycol to extend the coding segment away from the substrate. The detection probes were made by *in situ *synthesis using PGR (photogenerated reagent) chemistry. After RNA hybridization, tag-conjugating Cy3 dyes were circulated through the microfluidic chip for dye staining. Images were acquired using a GenePix 4000B (Molecular Device) laser scanner. Intensity data were collected using Array-Pro image analysis software (Media Cybernetics) with a scan resolution of 10 microns and PTM between 480 and 540 V. Analysis was conducted on background subtracted signal intensities. Background was determined using a regression-based background mapping method. Spots yielding true signal (i.e., present) had signal intensities higher than 3 ×(background standard deviation) and standard deviation/signal intensity < 0.5. CV is calculated by (standard deviation)/(signal intensity). Probes repeated multiple times on an array were called present if the signals from at least 50% of the probes were above detection level.

### Normalization and data processing

For two-color analyses, a dye swap was applied followed by a lowess normalization. For analysis in one color on Agilent, Exiqon and Invitrogen NCode arrays, non-background subtracted median signal intensities were cyclic-lowess normalized [28] in R [29]. Cyclic lowess normalization is a one colour normalization method which is a modification of lowess normalization for two colour studies. The method estimates a lowess curve for all distinct pairwise combinations. For all pairs of arrays for any array k where 1 = k = n, the adjustments for array k relative to the other arrays are averaged and applied to the raw data to obtain the normalized data [28]. The Cy3 channel was used for the Exiqon slides because Cy3 was used for LC Sciences and Agilent. The Alexa Fluor 5 channel was used for the NCode arrays because the Alexa Fluor 3 channel produced lower quality of data. The background for the Agilent array was measured using the (-)3xSLv1 probe, using the hsa_negative_control_1 probes for the Exiqon platform, and using the pool of random hexamers for NCode arrays. Spots with median signal intensities less than the trimmed mean plus three trimmed standard deviations of these negative controls were flagged as absent. A trimmed mean or truncated mean is a statistical measure of central tendency, where a percent of the high and low ends of a distribution are removed and the mean is calculated using the remaining data of the remaining distribution. In the present study the trim (or proportion of data removed) was set at 5%. MiRNA technical replicates were then averaged by taking the median of the technical replicates. The collapsed observation was flagged as absent if at least one of the values used to calculate the average was flagged.

Data processing of LC Sciences arrays was carried out by LC Sciences using their standard data analysis methods. Briefly, cyclic-lowess (locally-weighted regression) normalization was carried out on background-subtracted data according using the same methods as those described above and in [28]. Transcripts were considered to be present if the signal intensity was greater than 3 × (background standard deviation) and the spot CV < 0.5 (where CV = (standard deviation)/(signal intensity)).

Normalized and raw intensity values for all microarray data have been deposited in the NCBI Gene Expression Omnibus database under the accession number GSE19669.

### TaqMan arrays

Reference pools 1 and 2 were analyzed using TaqMan^® ^rodent microRNA arrays v2.0 (A and B) (Applied Biosystems). These arrays represent 585 mature miRNA derived from Sanger miRBase v10 analyzed over two 384-well PCR plates. RT-PCR reactions were carried out using the manufacturer's recommendation. In brief, 1 μg of total RNA was reverse transcribed using Megaplext RT Primers and TaqMan miRNA reverse transcription kids. Quantitative RT-PCR was performed at the University of Montreal http://www.iric.umontreal.ca/Recherche/Plates_Formes/Genomique_EN.html using an Applied Biosystems 7900HR system and TaqMan universal PCR master mix using the following conditions: 10 min at 94.5°C followed by 40 cycles of 97°C for 30 s and 59.7°C for 1 min. Cycle threshold (Ct) values were calculated using the SDS2.2.2 using the automatic baseline and threshold of 0.2. Normalized expression (NE) was calculated using NE = 2expDeltaDeltaCt, where Ct is the threshold cycle to detect fluorescence. The data were normalized to miRNA U6. Ct values that were greater than 35 were considered to be below the detection threshold of the assay [19].

### Filtering and statistical analysis

Because miRNA probes from the various platforms were based on different MiRBase databases (Table 1), probes were first mapped by sequence. This was a straightforward process relative to matching mRNA probes, as miRNA are very short. All the data were first merged using the name of the miRNA. Sequence matching was then carried out. The Agilent probe sequences were shorter than the probes from the other platforms. If the Agilent probe sequence was contained within a probe from the other platforms, it was considered a match.

Spearman correlations were determined for within-platform comparisons and for between-platform comparisons. Correlations were calculated separately for the set of miRNAs that were either 1) detectably expressed as 'present' on the two arrays being compared, or 2) detectably expressed on all four array platforms and gave measurable Ct values using RT-PCR.

Within platform correlations were conducted either using all miRNAs detectably expressed as present on that platform, or using the miRNAs that were present on all four platforms and gave measurable Ct values using RT-PCR. The Spearman correlation between log_2_(Ref. 1_A_/Ref. 2_A_) vs. log_2_(Ref. 1_B_/Ref. 2_B_) and log_2_(Ref. 1_A_/Ref. 2_B_) vs. log_2_(Ref. 1_B_/Ref. 2_A_) were averaged, and standard error and 95% confidence intervals were obtained using the bootstrap [30].

Spearman correlations between the mean relative differences (log_2_) for each platform to the relative difference obtained from RT-PCR were estimated. Standard Errors and 95% confidence intervals were obtained as above. Similarly, for comparisons between different array platforms, Spearman correlations with standard errors and 95% confidence intervals for mean relative differences (log_2_) were also estimated. Similar to the within-platform correlations, all correlations were determined based on either all miRNAs that were detectably expressed on the two platforms being compared, or all miRNAs that were detectably expressed on all four array platforms, plus the RT-PCR arrays. The Concordance correlation coefficient [23] (CCC) analysis was conducted using the epiR R library 2 [31].

Finally, two of the platforms evaluated were one-color arrays (Agilent, LC Science) with two platforms running two-color arrays (Exiqon, Invitrogen NCode). To evaluate whether this distinction led to increased differences between platforms, each of the two-color arrays were analyzed both as a two-color arrays, and as a one-color array.

## List of Abbreviations

miRNA: microRNA

## Authors' contributions

The concept of the experiment and the experiment design were conceived by CLY and AW. Sample preparation and miRNA analysis with Agilent and Exiqon platforms were carried out by AR-C. NCode arrays were printed and hybridized by JDHS. The arrays were sequence matched data, filtered and normalized by AW, who also performed the correlation analysis. The manuscript was written by CLY with input from all authors. All authors have read and approved the final manuscript.

## Supplementary Material

Additional file 1 **Number of miRNAs in common (from Present miRNAs only; 2-color data)**. A Venn diagram depicting the overlap of miRNA probes in common across the platforms for the 2-color data. Note that the overall number of miRNAs in common (54) would be the overlap of the intersects of these two Venn diagrams. Please also note that the present calls for one color analysis will be different from 2-color because the data processing is slightly different for the 2 approaches (i.e., there are twice as many data points in the two color analysis [dye swap], and the data processing involved in the 1 versus 2 color introduces additional variation associated with present or absent calls). Click here for file

Additional file 2 **Venn diagram depicting the number of miRNAs in common (from Present miRNAs only; 1-color data)**. A Venn diagram is shown for the overlap of miRNA probes in common across the platforms for the 1-colour analysis. Note that the overall number of miRNAs in common (54) would be the overlap of the intersects of these two Venn diagrams. Please also note that the present calls for one color analysis, will be different from 2-color because the data processing is slightly different for the 2 approaches (i.e., there are twice as many data points in the two color analysis [dye swap], and the data processing involved in the 1 versus 2 color introduces additional variation associated with present or absent calls). Click here for file

Additional file 3 **Individual Spearman rank correlations**. Summary of all the individual Spearman rank correlations conducted. R1.1 = Reference 1, replicate 1. R2.1 = Reference 2, replicate 1. R1.2 = Reference 1, replicate 2. R2.1 = Reference 2, replicate 1. Click here for file

Additional file 4 **Scatterplots for all pair-wise comparisons**. Scatterplots for all pair-wise comparisons of the log_2 _ratio of Pool A to Pool B are presented for each platform. Present miRNAs are in red, while miRNAs that were called 'absent' (i.e., not a high enough signal) are depicted in blue. The correlation coefficients (R^2^), slopes and intercepts are shown in the top left corner of the figure for the 'Present' miRNAs, and bottom right corner for the 'Absent' miRNAs. Click here for file

Additional file 5 **Correlation of platforms with Taqman RT-PCR**. A permutation test was used to test the correlation of platforms with Taqman RT-PCR (i.e., compare accuracy between platforms). The p-value for testing differences between the correlation coefficients was calculated using permutation analysis. Here, 5000 permutations were used to generate the null distribution of the test statistic. Click here for file

## References

[B1] Singh SK Pal Bhadra M Girschick HJ Bhadra U MicroRNAs--micro in size but macro in function FEBS J 2008 275 20 4929 4944 10.1111/j.1742-4658.2008.06624.x 18754771

[B2] Carleton M Cleary MA Linsley PS MicroRNAs and cell cycle regulation Cell Cycle 2007 6 17 2127 2132 17786041 10.4161/cc.6.17.4641

[B3] Croce CM Causes and consequences of microRNA dysregulation in cancer Nat Rev Genet 2009 10 10 704 714 10.1038/nrg2634 19763153 PMC3467096

[B4] Hudder A Novak RF miRNAs: effectors of environmental influences on gene expression and disease Toxicol Sci 2008 103 2 228 240 10.1093/toxsci/kfn033 18281715 PMC2922762

[B5] Lu J Getz G Miska EA Alvarez-Saavedra E Lamb J Peck D Sweet-Cordero A Ebert BL Mak RH Ferrando AA Downing JR Jacks T Horvitz HR Golub TR MicroRNA expression profiles classify human cancers Nature 2005 435 7043 834 838 10.1038/nature03702 15944708

[B6] Wang K Zhang S Marzolf B Troisch P Brightman A Hu Z Hood LE Galas DJ Circulating microRNAs, potential biomarkers for drug-induced liver injury Proc Natl Acad Sci USA 2009 106 11 4402 4407 10.1073/pnas.0813371106 19246379 PMC2657429

[B7] Wang Y Liang Y Lu Q MicroRNA epigenetic alterations: predicting biomarkers and therapeutic targets in human diseases Clin Genet 2008 74 4 307 315 10.1111/j.1399-0004.2008.01075.x 18713257

[B8] Xiao C Rajewsky K MicroRNA control in the immune system: basic principles Cell 2009 136 1 26 36 10.1016/j.cell.2008.12.027 19135886

[B9] Ach RA Wang H Curry B Measuring microRNAs: comparisons of microarray and quantitative PCR measurements, and of different total RNA prep methods BMC Biotechnol 2008 8 69 10.1186/1472-6750-8-69 18783629 PMC2547107

[B10] Chen Y Gelfond JA McManus LM Shireman PK Reproducibility of quantitative RT-PCR array in miRNA expression profiling and comparison with microarray analysis BMC Genomics 2009 10 407 10.1186/1471-2164-10-407 19715577 PMC2753550

[B11] Sato F Tsuchiya S Terasawa K Tsujimoto G Intra-platform repeatability and inter-platform comparability of microRNA microarray technology PLoS One 2009 4 5 e5540 10.1371/journal.pone.0005540 19436744 PMC2677665

[B12] Yauk CL Berndt ML Review of the literature examining the correlation among DNA microarray technologies Environ Mol Mutagen 2007 48 5 380 394 10.1002/em.20290 17370338 PMC2682332

[B13] Chen JJ Hsueh HM Delongchamp RR Lin CJ Tsai CA Reproducibility of microarray data: a further analysis of microarray quality control (MAQC) data BMC Bioinformatics 2007 8 412 10.1186/1471-2105-8-412 17961233 PMC2204045

[B14] Shi L Tong W Fang H Scherf U Han J Puri RK Frueh FW Goodsaid FM Guo L Su Z Han T Fuscoe JC Xu ZA Patterson TA Hong H Xie Q Perkins RG Chen JJ Casciano DA Cross-platform comparability of microarray technology: intra-platform consistency and appropriate data analysis procedures are essential BMC Bioinformatics 2005 6 Suppl 2 S12 10.1186/1471-2105-6-S2-S12 16026597 PMC1637032

[B15] Stafford P Brun M Three methods for optimization of cross-laboratory and cross-platform microarray expression data Nucleic Acids Res 2007 35 10 e72 10.1093/nar/gkl1133 17478523 PMC1904274

[B16] Yauk CL Berndt ML Williams A Douglas GR Comprehensive comparison of six microarray technologies Nucleic Acids Res 2004 32 15 e124 10.1093/nar/gnh123 15333675 PMC516080

[B17] Nelson PT Wang WX Wilfred BR Tang G Technical variables in high-throughput miRNA expression profiling: much work remains to be done Biochim Biophys Acta 2008 1779 11 758 765 18439437 10.1016/j.bbagrm.2008.03.012 PMC2660892

[B18] Irizarry RA Warren D Spencer F Kim IF Biswal S Frank BC Gabrielson E Garcia JG Geoghegan J Germino G Griffin C Hilmer SC Hoffman E Jedlicka AE Kawasaki E Martinez-Murillo F Morsberger L Lee H Petersen D Quackenbush J Scott A Wilson M Yang Y Ye SQ Yu W Multiple-laboratory comparison of microarray platforms Nat Methods 2005 2 5 345 350 10.1038/nmeth756 15846361

[B19] Guthrie JL Seah C Brown S Tang P Jamieson F Drews SJ Use of Bordetella pertussis BP3385 to establish a cutoff value for an IS481-targeted real-time PCR assay J Clin Microbiol 2008 46 11 3798 3799 10.1128/JCM.01551-08 18784312 PMC2576621

[B20] Guo L Lobenhofer EK Wang C Shippy R Harris SC Zhang L Mei N Chen T Herman D Goodsaid FM Hurban P Phillips KL Xu J Deng X Sun YA Tong W Dragan YP Shi L Rat toxicogenomic study reveals analytical consistency across microarray platforms Nat Biotechnol 2006 24 9 1162 1169 10.1038/nbt1238 17061323

[B21] Shi L Rei LH Jones WD Shippy R Warrington JA Baker SC Collins PJ de Longueville F Kawasaki ES Lee KY Luo Y Sun YA Willey JC Setterquist RA Fischer GM Tong W Dragan YP Dix DJ Frueh FW Goodsaid FM Herman D Jensen RV Johnson CD Lobenhofer EK Puri RK Schrf U Thierry-Mieg J Wang C Wilson M Wolber PK The MicroArray Quality Control (MAQC) project shows inter- and intraplatform reproducibility of gene expression measurements Nat Biotechnol 2006 24 9 1151 1161 10.1038/nbt1239 16964229 PMC3272078

[B22] Lin LI A concordance correlation coefficient to evaluate reproducibility Biometrics 1989 45 1 255 268 10.2307/2532051 2720055

[B23] Miron M Woody OZ Marcil A Murie C Sladek R Nadon R A methodology for global validation of microarray experiments BMC Bioinformatics 2006 7 333 10.1186/1471-2105-7-333 16822306 PMC1539027

[B24] Sah S McCall MN Eveleigh D Wilson M Irizarry RA Performance evaluation of commercial miRNA expression array platforms BMC Res Notes 2010 3 80 10.1186/1756-0500-3-80 20298588 PMC2853548

[B25] Yauk CL Williams A Boucher S Berndt LM Zhou G Zheng JL Rowan-Carroll A Dong H Lambert IB Douglas GR Parfett CR Novel design and controls for focused DNA microarrays: applications in quality assurance/control and normalization for the Health Canada ToxArray BMC Genomics 2006 7 266 10.1186/1471-2164-7-266 17052352 PMC1635050

[B26] Gao X Gulari E Zhou X In situ synthesis of oligonucleotide microarrays Biopolymers 2004 73 579 596 10.1002/bip.20005 15048782

[B27] Zhu Q Hong A Sheng N Zhang X Jun K-Y Srivannavit O Gulari E Gao X Zhou X Rampal JB Microfluidic biochip for nucleic acid and protein analysis Methods Mol Biol 2007 382 287 312 full_text 18220239 10.1007/978-1-59745-304-2_19

[B28] Bolstad BM Irizarry RA Astrand M Speed TP A comparison of normalization methods for high density oligonucleotide array data based on variance and bias Bioinformatics 2003 19 2 185 193 10.1093/bioinformatics/19.2.185 12538238

[B29] R.D.C. T: R A language and environment for statistical computing 2004 http://www.r-project.org/

[B30] Zhu D Li Y Li H Multivariate correlation estimator for inferring functional relationships from replicated genome-wide data Bioinformatics 2007 23 17 2298 2305 10.1093/bioinformatics/btm328 17586543

[B31] Stevenson S Nunes T Sanchez J Thornton R epiR: Functions for analysing epidemiological data R package version 0.9-22 2009 http://CRAN.R-project.org/package=epiR

