# Supplementary figures and images for: Cross-platform analysis of global microRNA expression technologies
Source: BMC Genomics. 2010 May 26;11:330. doi: 10.1186/1471-2164-11-330 (PMC2890562; doi:10.1186/1471-2164-11-330)

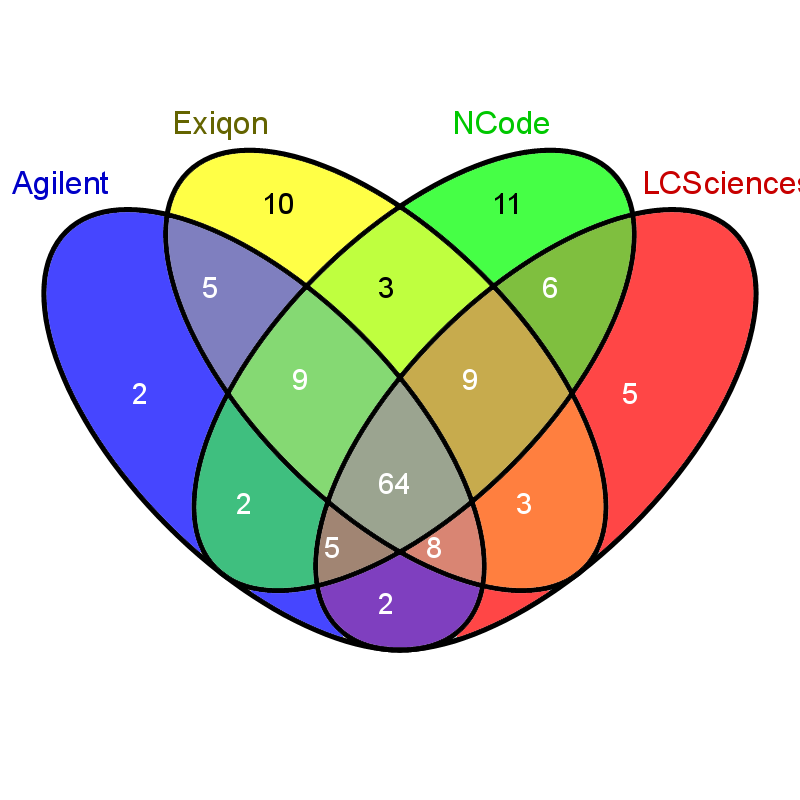

Supplement: Additional file 1 — Number of miRNAs in common (from Present miRNAs only; 2-color data). A Venn diagram depicting the overlap of miRNA probes in common across the platforms for the 2-color data. Note that the overall number of miRNAs in common (54) would be the overlap of the intersects of these two Venn diagrams. Please also note that the present calls for one color analysis will be different from 2-color because the data processing is slightly different for the 2 approaches (i.e., there are twice as many data points in the two color analysis [dye swap], and the data processing involved in the 1 versus 2 color introduces additional variation associated with present or absent calls). [file 1471-2164-11-330-S1.PNG]

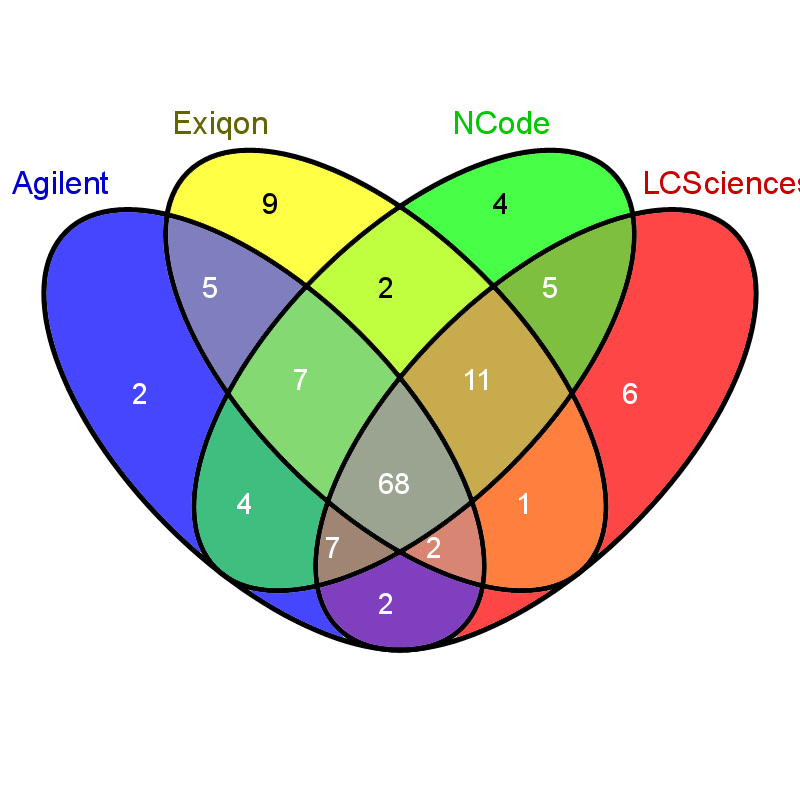

Supplement: Additional file 2 — Venn diagram depicting the number of miRNAs in common (from Present miRNAs only; 1-color data). A Venn diagram is shown for the overlap of miRNA probes in common across the platforms for the 1-colour analysis. Note that the overall number of miRNAs in common (54) would be the overlap of the intersects of these two Venn diagrams. Please also note that the present calls for one color analysis, will be different from 2-color because the data processing is slightly different for the 2 approaches (i.e., there are twice as many data points in the two color analysis [dye swap], and the data processing involved in the 1 versus 2 color introduces additional variation associated with present or absent calls). [file 1471-2164-11-330-S2.PNG]

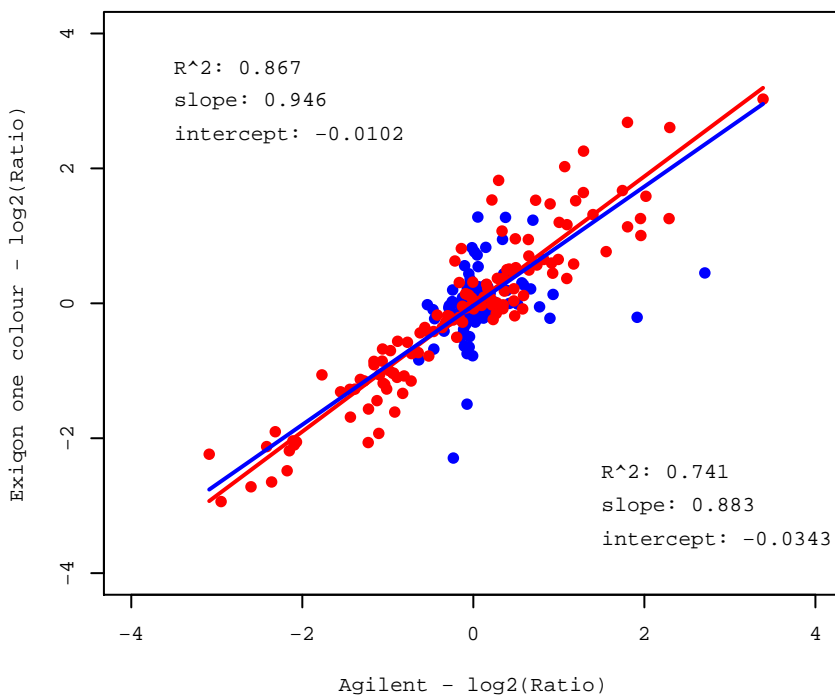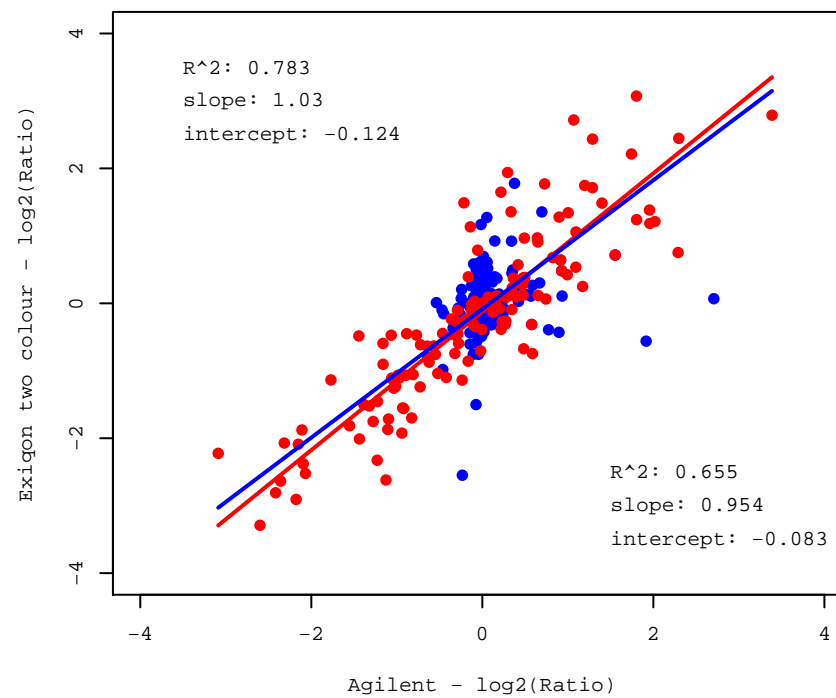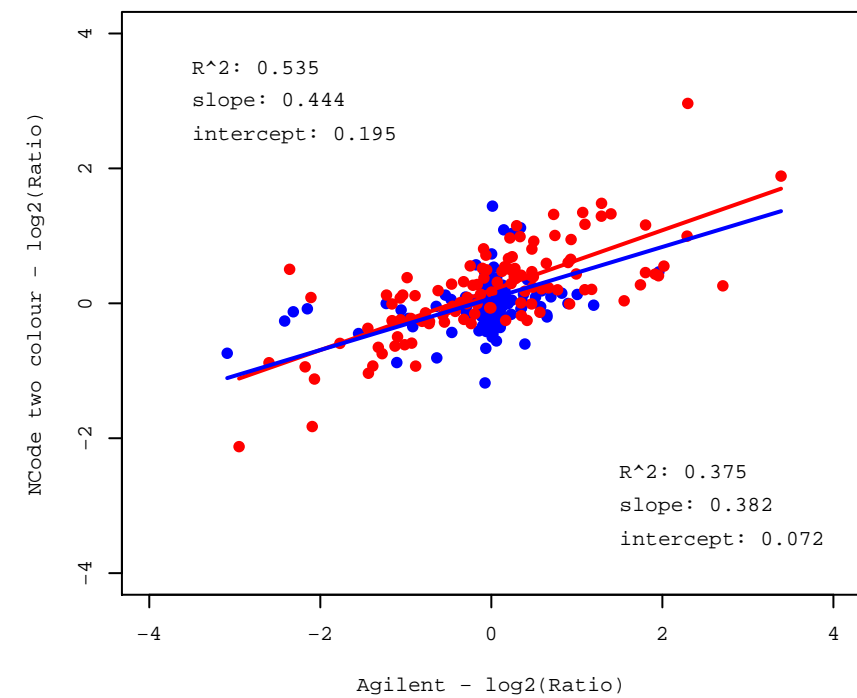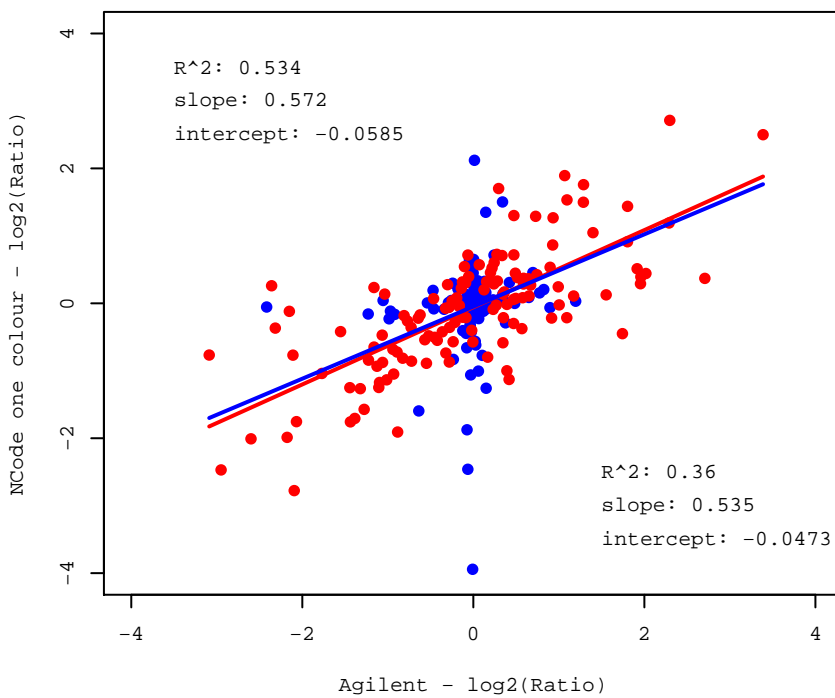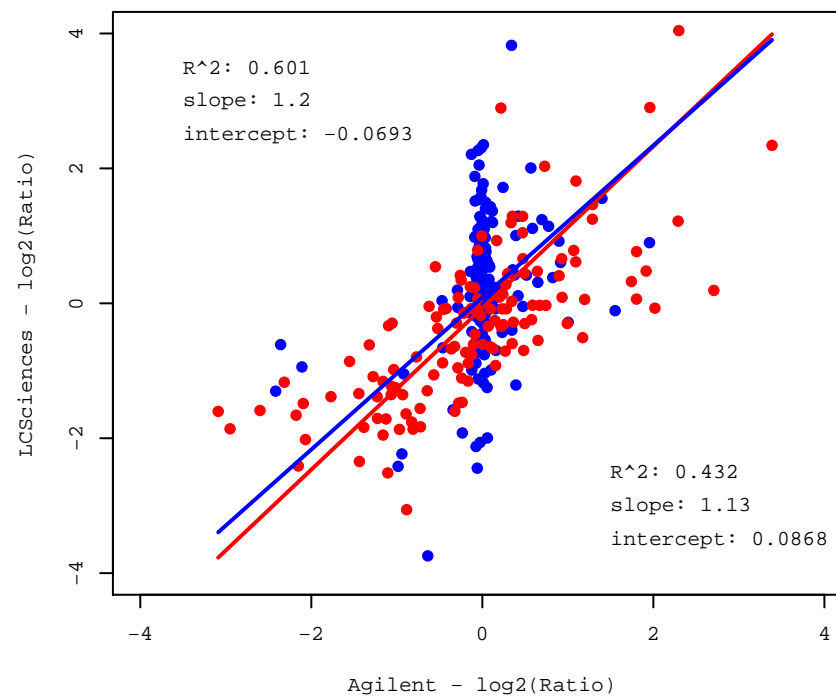

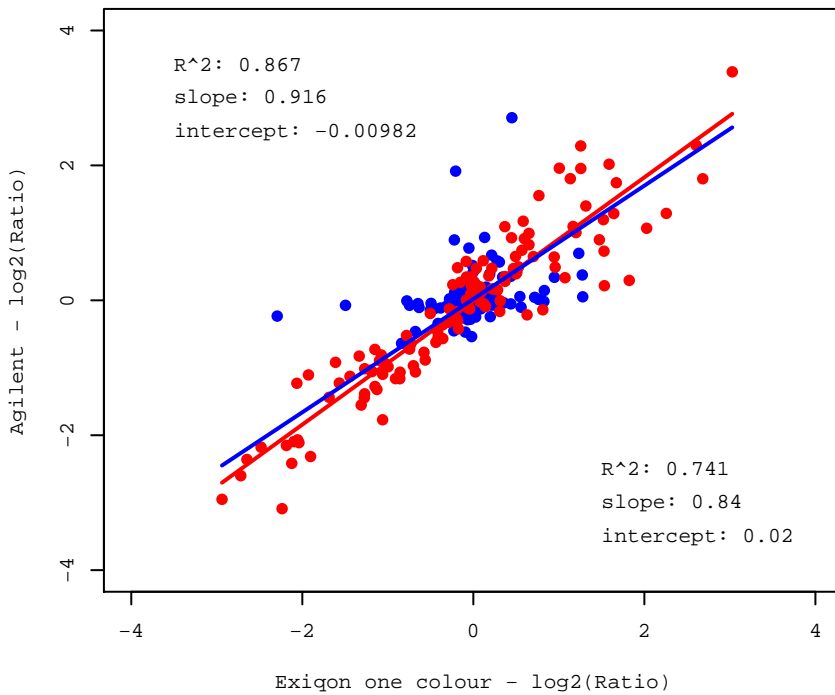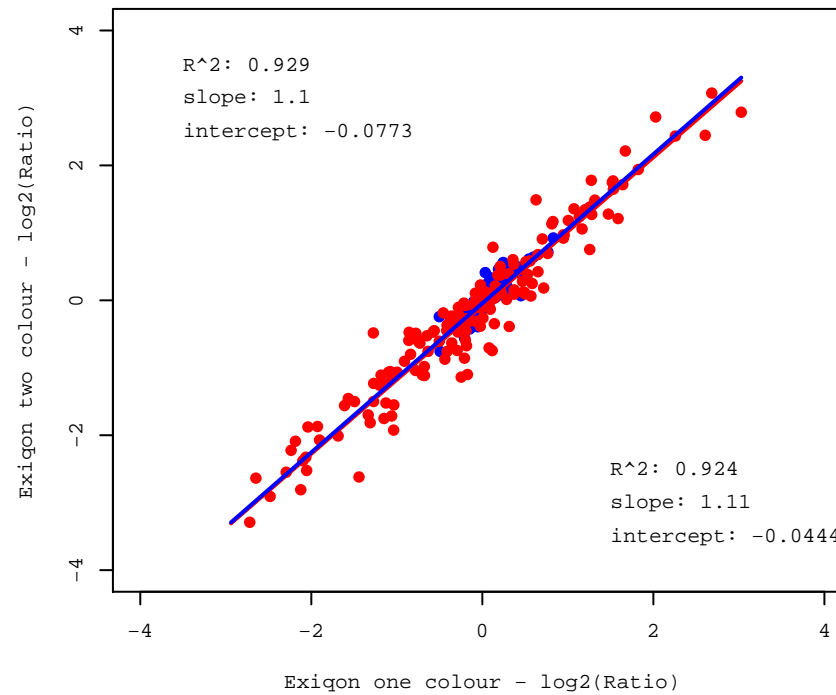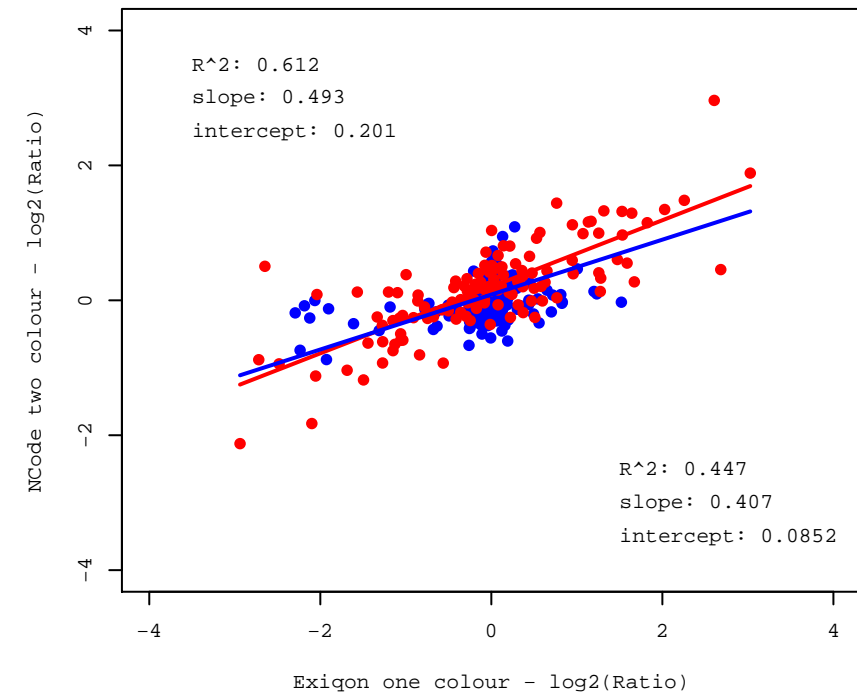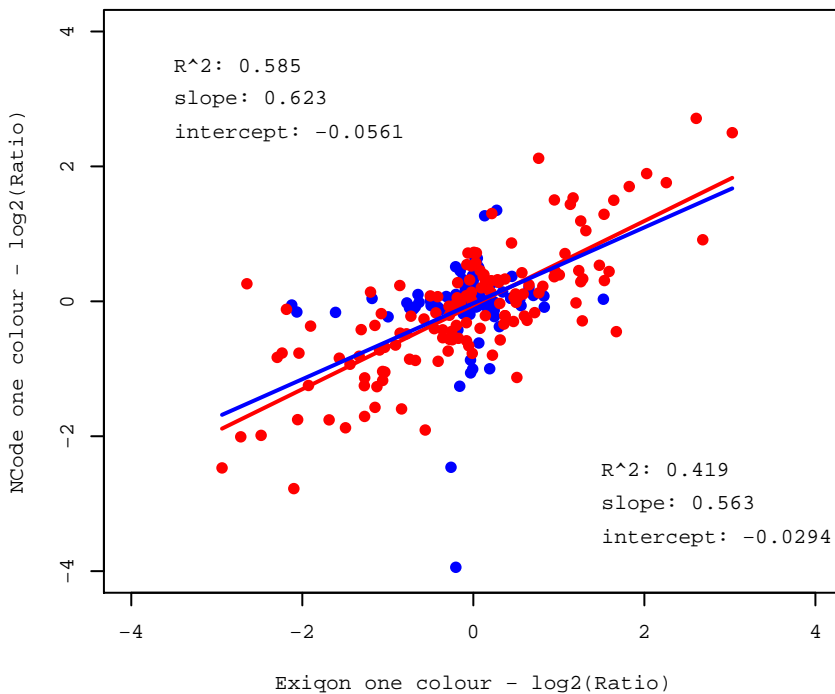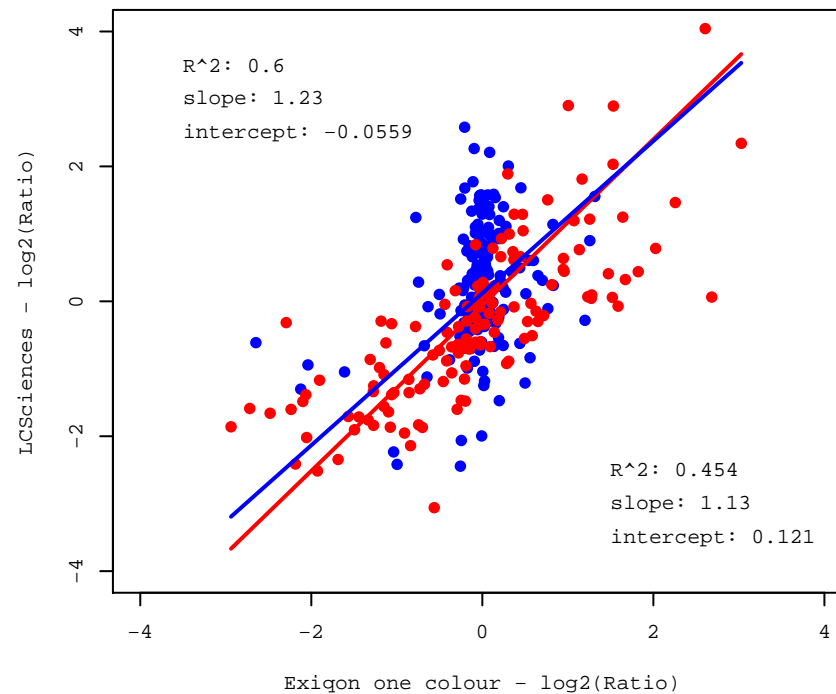

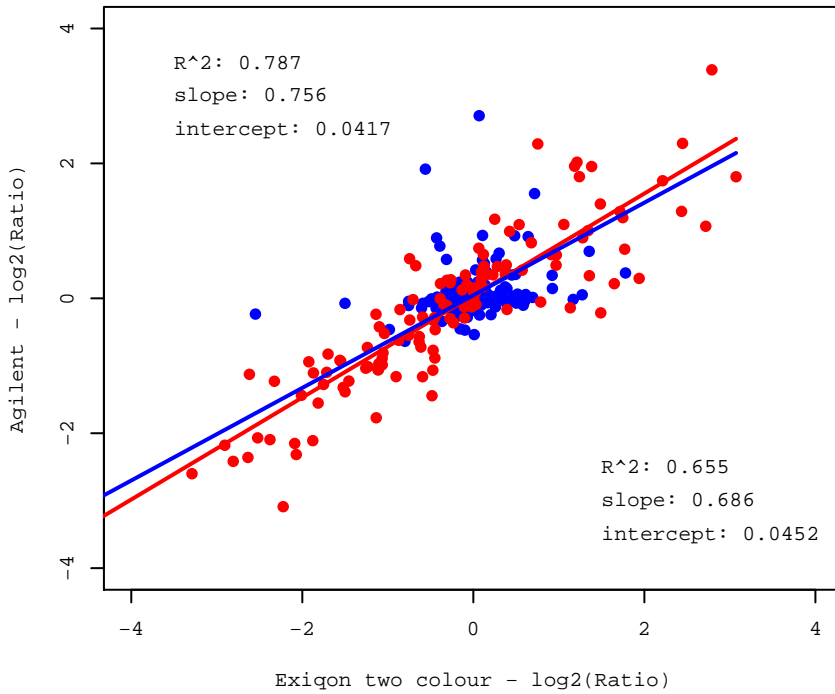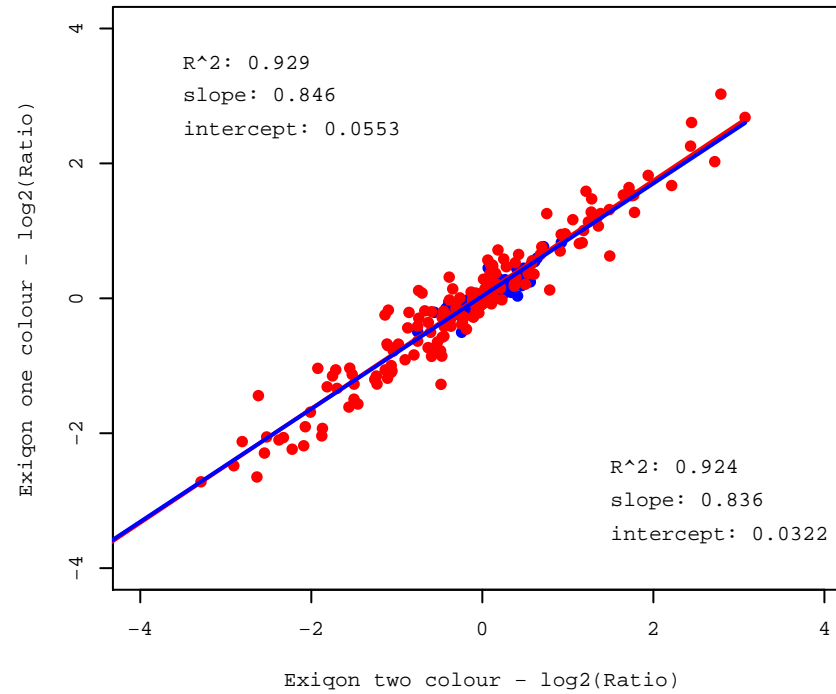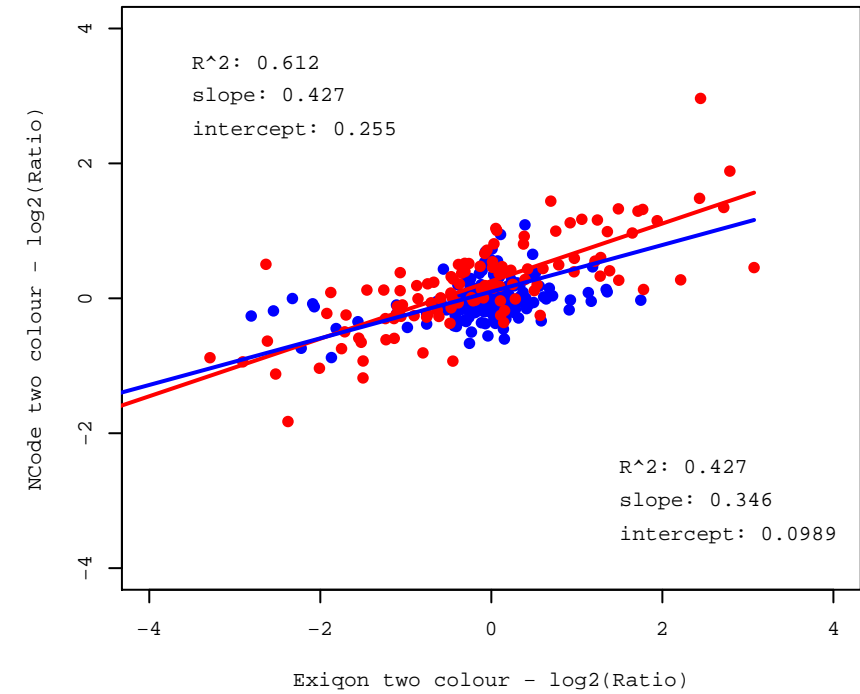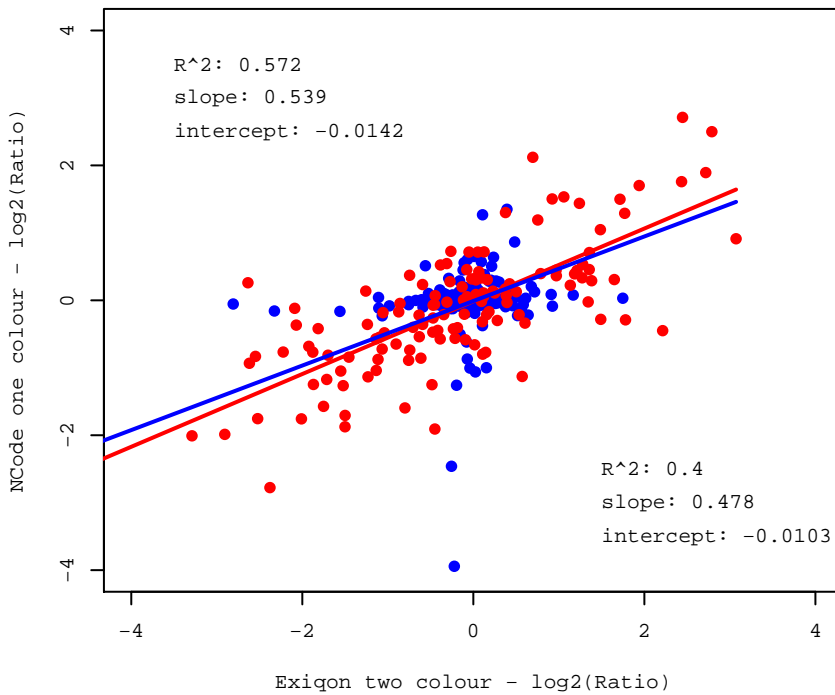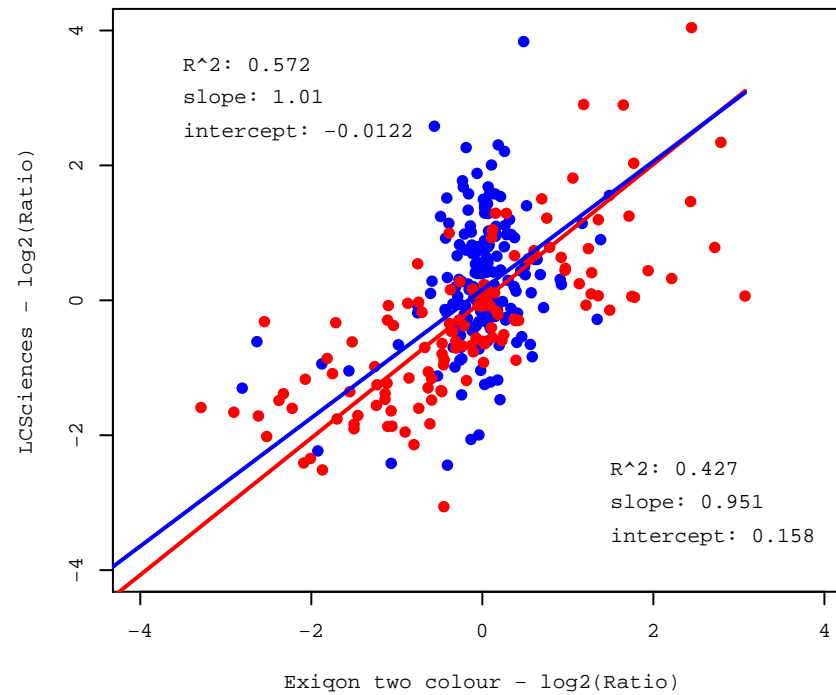

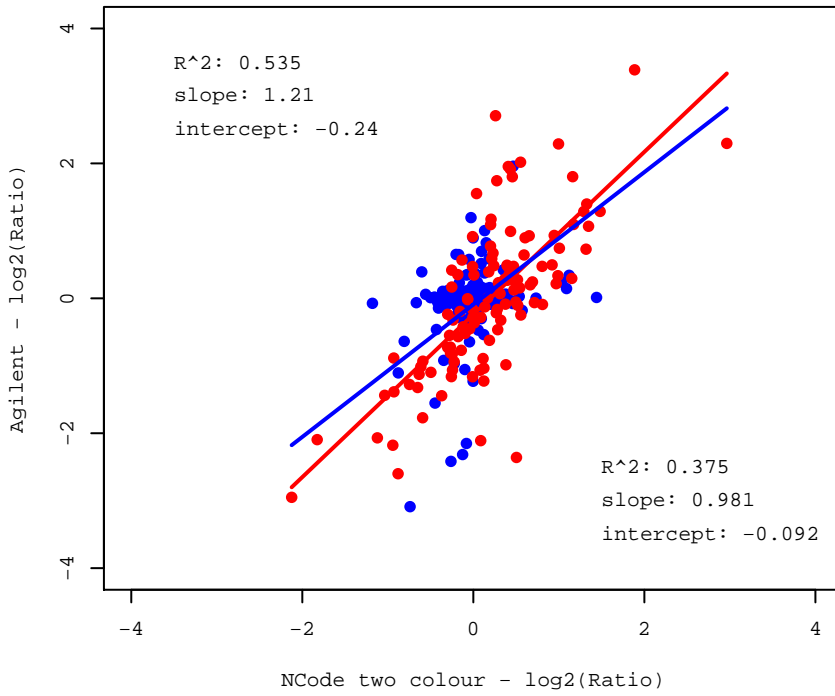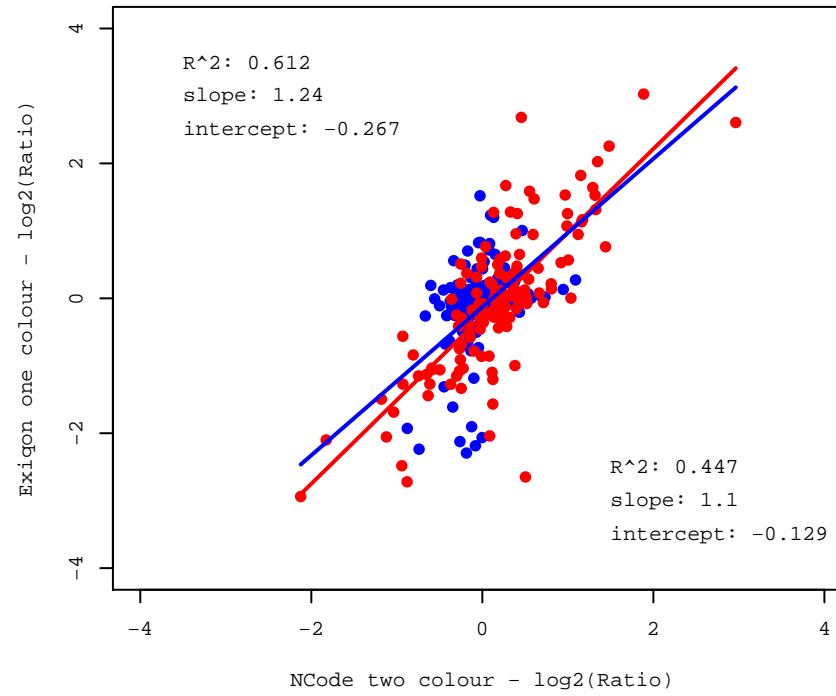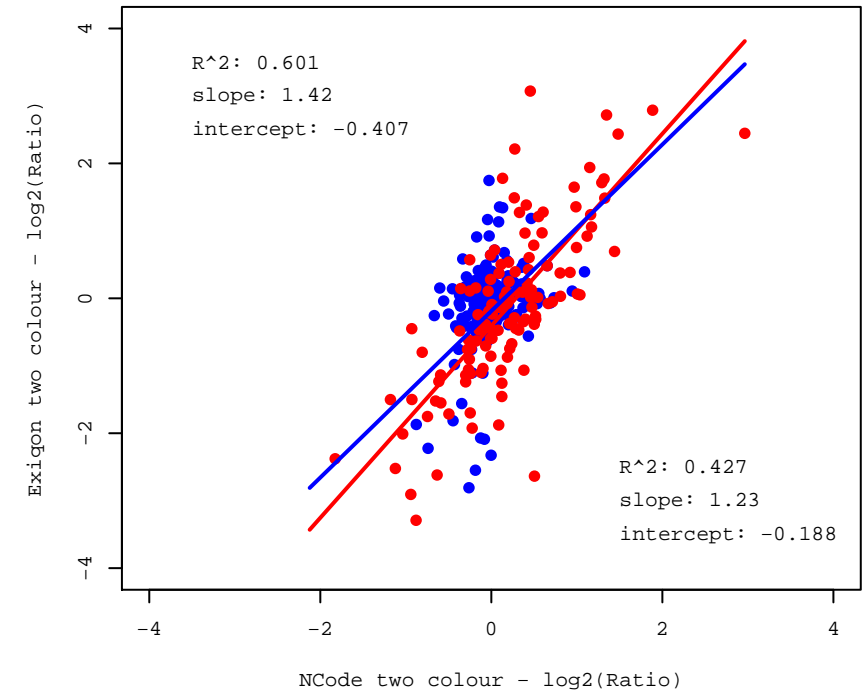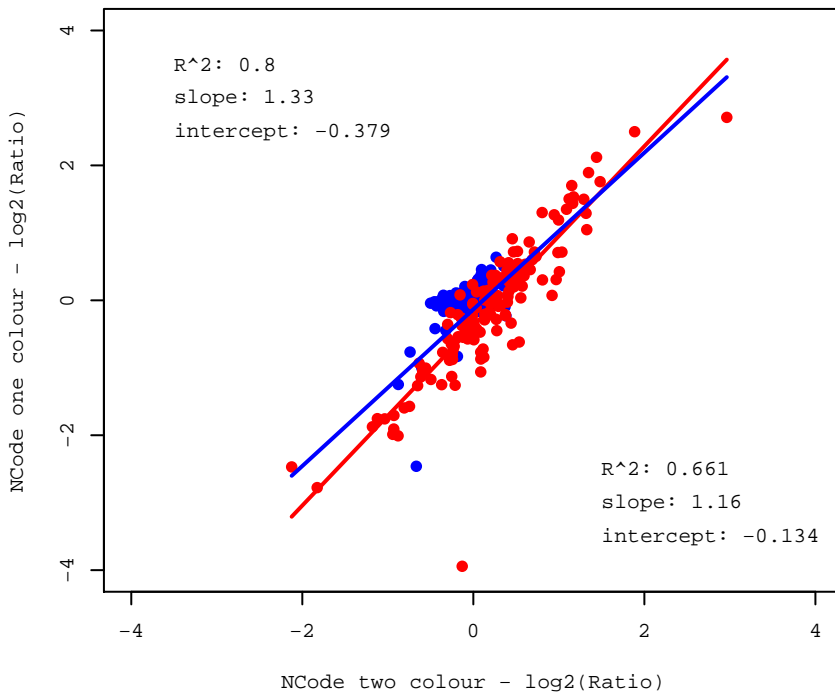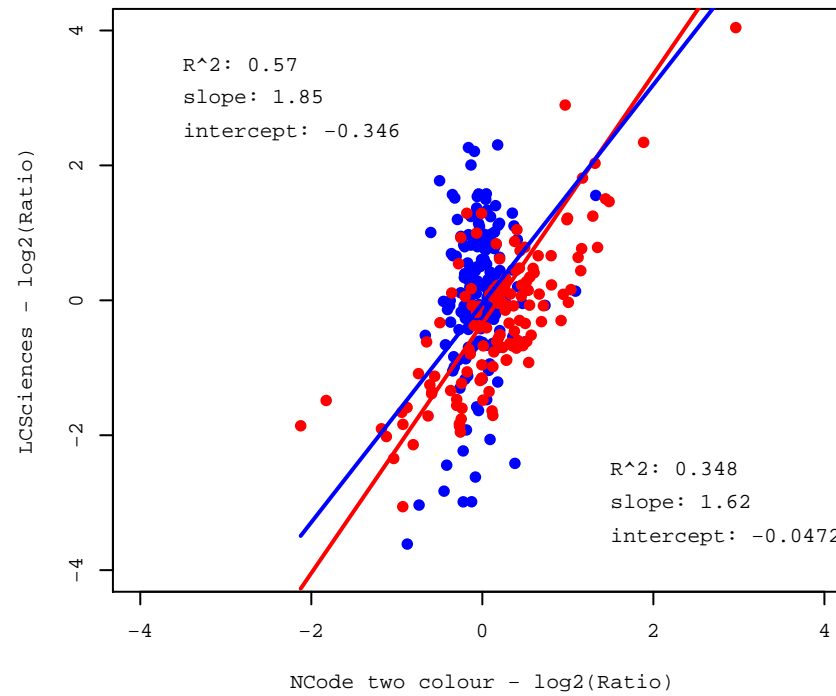

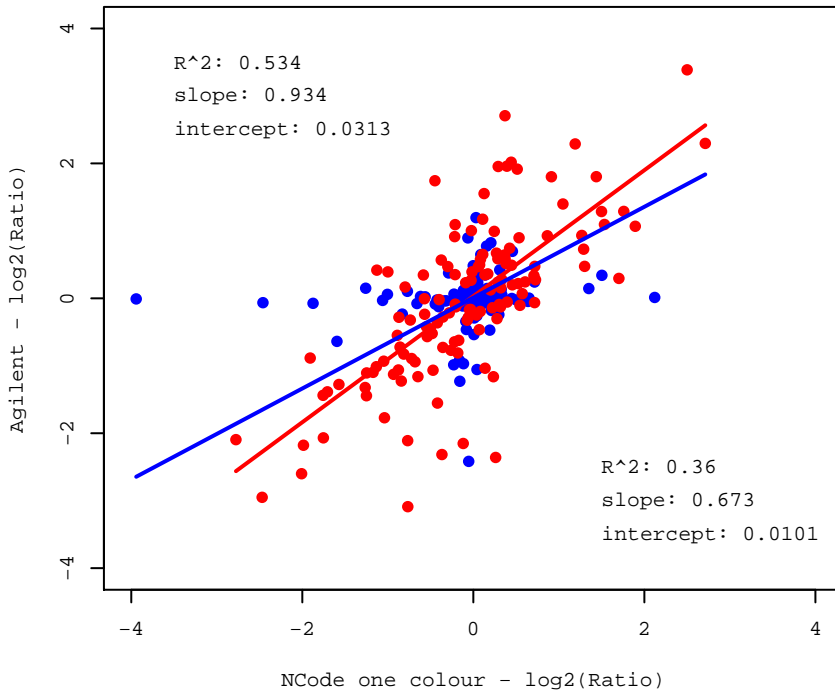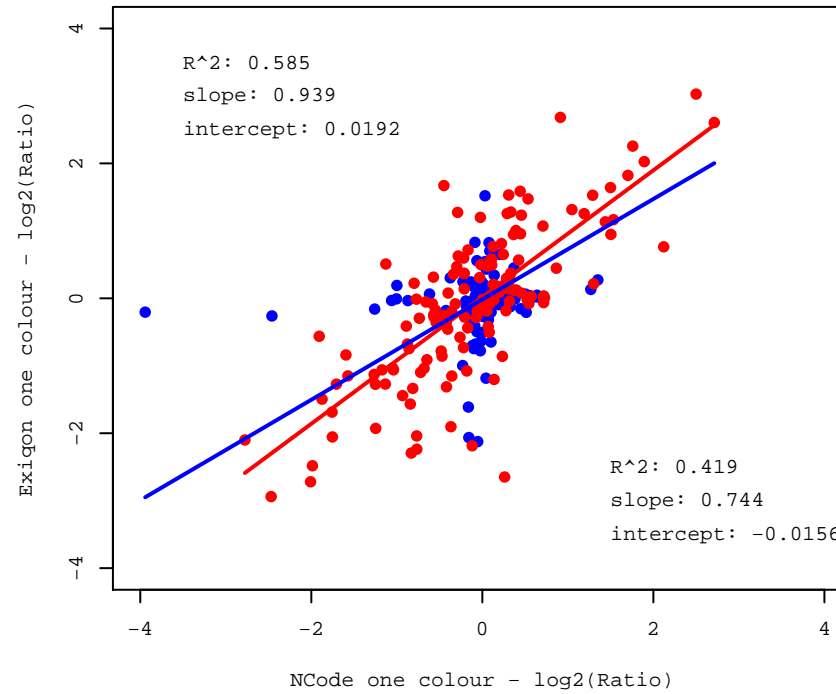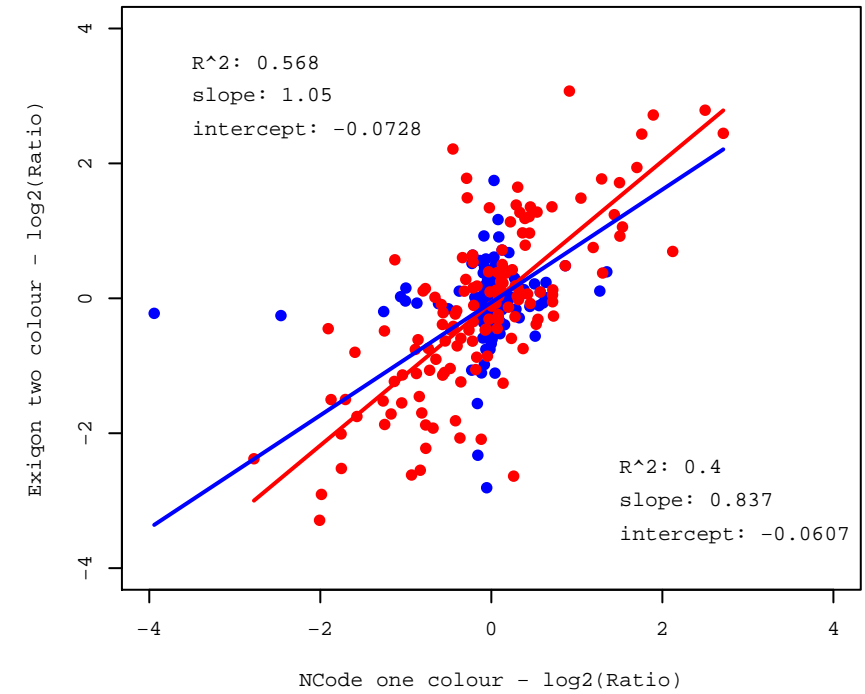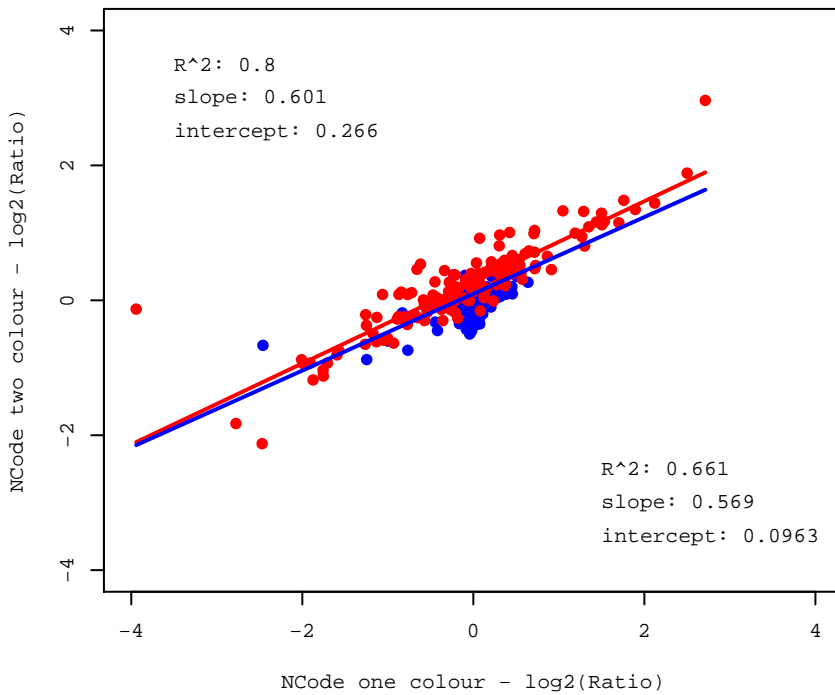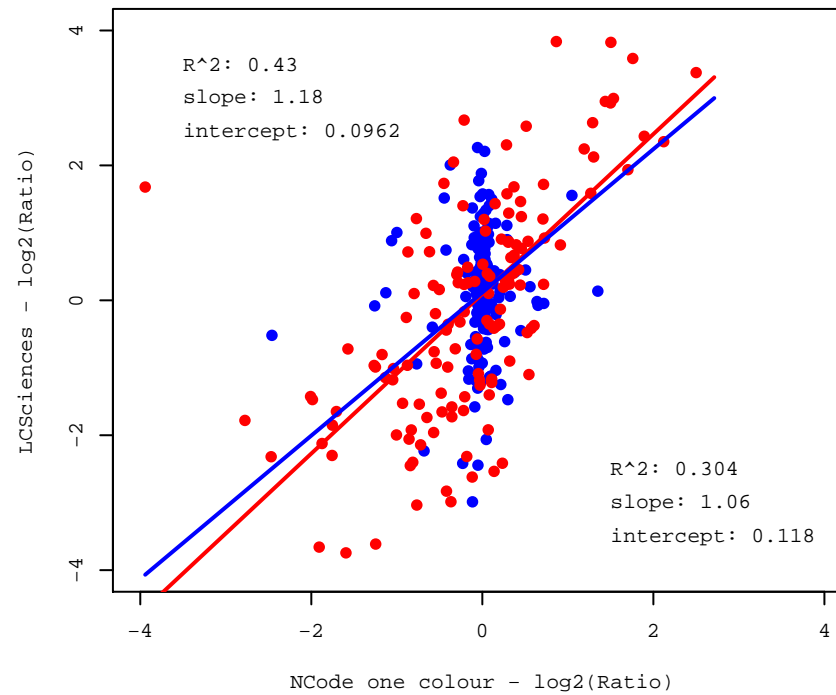

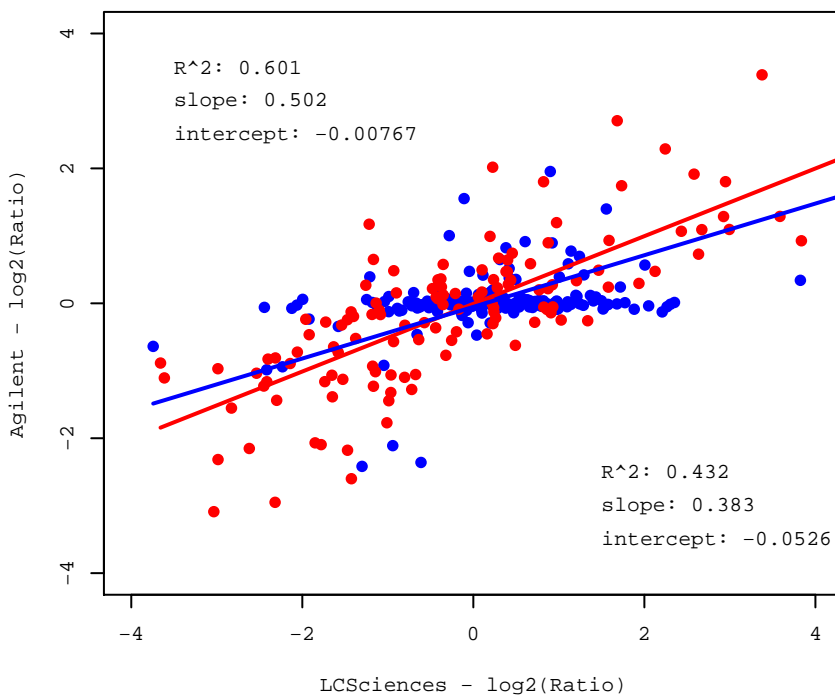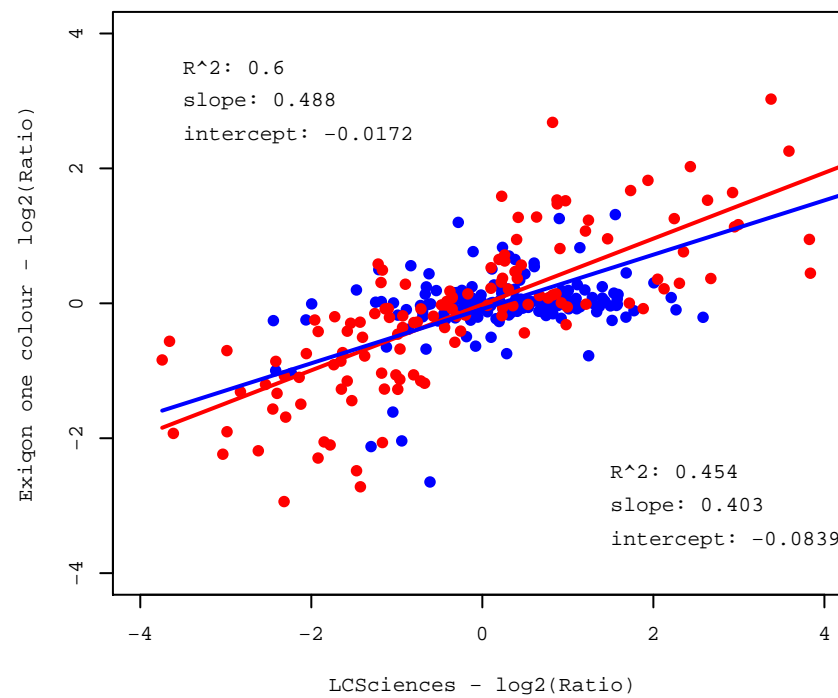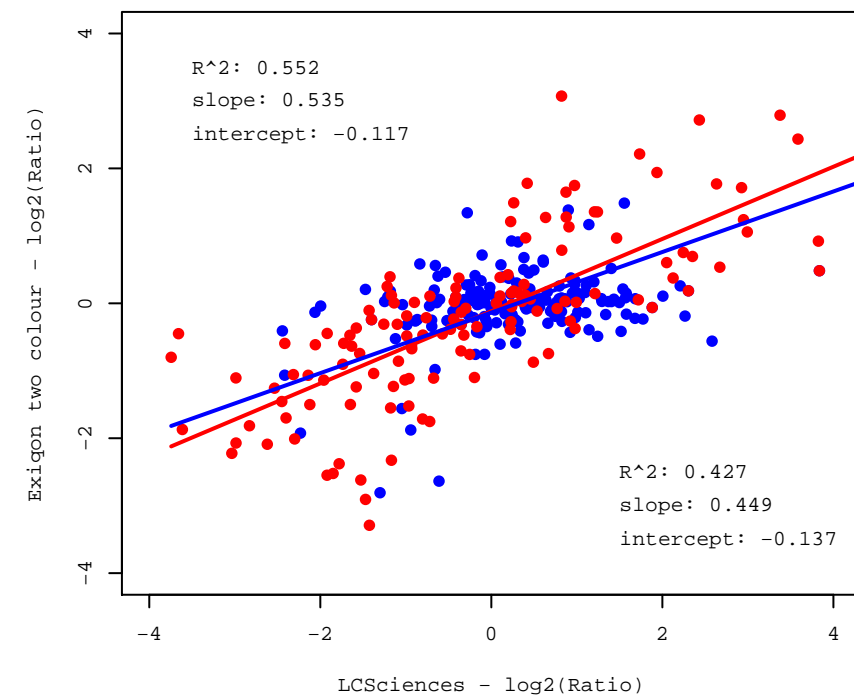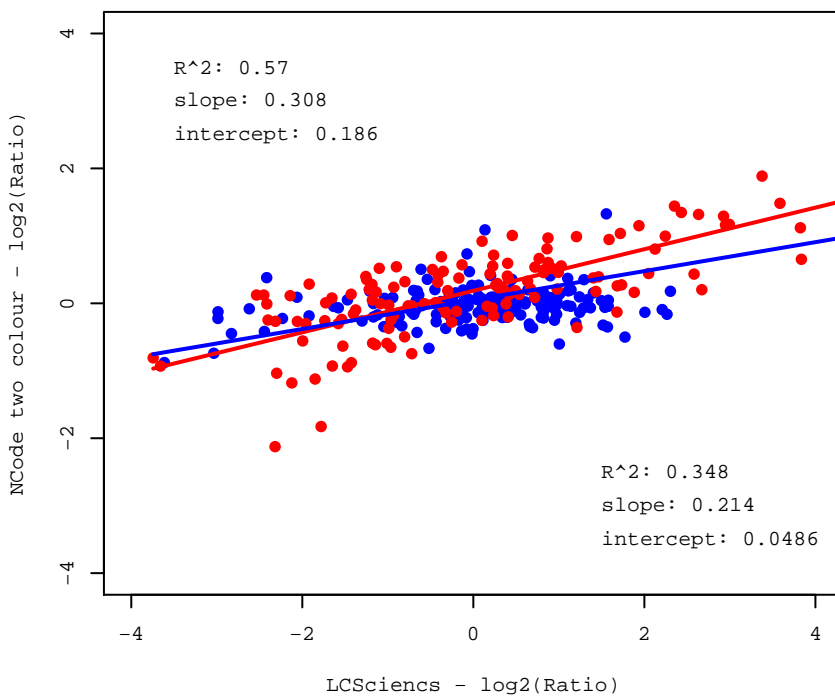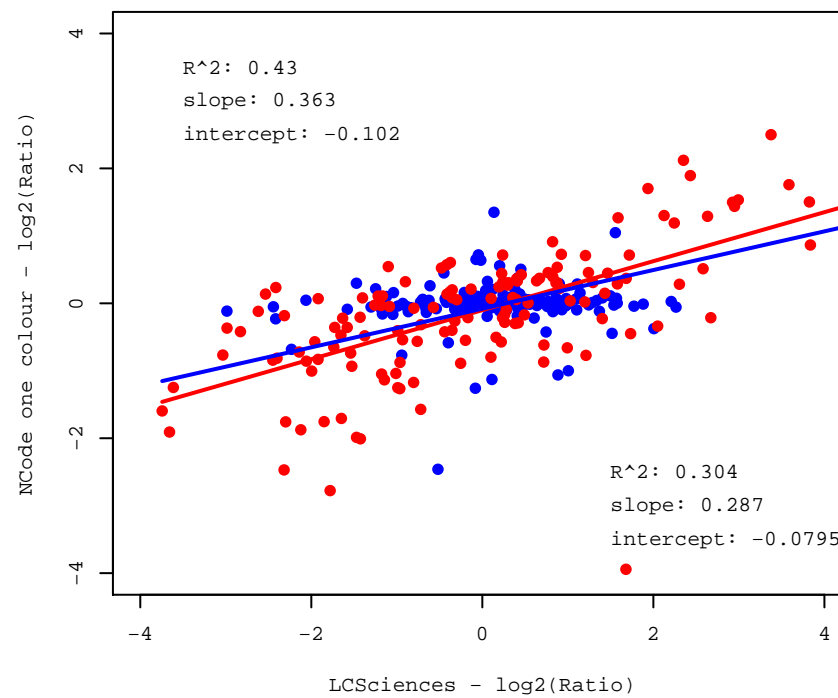

Supplement: Additional file 4 — Scatterplots for all pair-wise comparisons. Scatterplots for all pair-wise comparisons of the log2 ratio of Pool A to Pool B are presented for each platform. Present miRNAs are in red, while miRNAs that were called 'absent' (i.e., not a high enough signal) are depicted in blue. The correlation coefficients (R2), slopes and intercepts are shown in the top left corner of the figure for the 'Present' miRNAs, and bottom right corner for the 'Absent' miRNAs. [file 1471-2164-11-330-S4.PDF]
