# Supplementary material for: Cross-platform analysis of global microRNA expression technologies
Source: BMC Genomics. 2010 May 26;11:330. doi: 10.1186/1471-2164-11-330 (PMC2890562; doi:10.1186/1471-2164-11-330)
Supplement: Additional file 3 — Individual Spearman rank correlations. Summary of all the individual Spearman rank correlations conducted. R1.1 = Reference 1, replicate 1. R2.1 = Reference 2, replicate 1. R1.2 = Reference 1, replicate 2. R2.1 = Reference 2, replicate 1. [file 1471-2164-11-330-S3.DOC]

**Table S1.**

|  | 54 Sequenced Matched miRNAs and Present on all Platforms | | Present Sequenced  Matched miRNAs | | |
| --- | --- | --- | --- | --- | --- |
| Individual Correlations Within Platforms | R.1.1/R.2.1 vs R.1.2/R.2.2 | R.1.1/R.2.2 vs R.1.2/R.2.1 | Number of Probes | R.1.1/R.2.1 vs R.1.2/R.2.2 | R.1.1/R.2.2 vs R.1.2/R.2.1 |
|  |  |  |  |  |  |
| Agilent | 0.995 | 0.995 | 122 | 0.996 | 0.995 |
| Exiqon 2 color | 0.987 | 0.838 | 131 | 0.984 | 0.845 |
| Exiqon 1 color | 0.795 | 0.809 | 125 | 0.872 | 0.876 |
| NCode 2 color | 0.863 | 0.769 | 118 | 0.778 | 0.633 |
| Ncode 1 color | 0.865 | 0.836 | 124 | 0.849 | 0.857 |
| LC Sciences | 0.992 | 0.970 | 131 | 0.979 | 0.977 |
