# Supplementary material for: Cross-platform analysis of global microRNA expression technologies
Source: BMC Genomics. 2010 May 26;11:330. doi: 10.1186/1471-2164-11-330 (PMC2890562; doi:10.1186/1471-2164-11-330)
Supplement: Additional file 5 — Correlation of platforms with Taqman RT-PCR. A permutation test was used to test the correlation of platforms with Taqman RT-PCR (i.e., compare accuracy between platforms). The p-value for testing differences between the correlation coefficients was calculated using permutation analysis. Here, 5000 permutations were used to generate the null distribution of the test statistic. [file 1471-2164-11-330-S5.DOC]

**Table S2.**

| Comparison | Correlation 1 | Correlation 2 | Difference | *p-*value |
| --- | --- | --- | --- | --- |
| Agilent vs Exiqon one color | 0.653 | 0.691 | -0.038 | 0.4100 |
| Agilent vs Exiqon two color | 0.653 | 0.656 | -0.003 | 0.9354 |
| Agilent vs LC Sciences | 0.653 | 0.506 | 0.148 | 0.1156 |
| Agilent vs NCode one color | 0.653 | 0.730 | -0.076 | 0.3046 |
| Agilent vs NCode two color | 0.653 | 0.775 | -0.122 | 0.0992 |
| Exiqon one color vs LC Sciences | 0.691 | 0.506 | 0.186 | 0.0280 |
| Exiqon one color vs NCode one color | 0.691 | 0.730 | -0.038 | 0.6452 |
| Exiqon one color vs NCode two color | 0.691 | 0.775 | -0.084 | 0.2952 |
| Exiqon two color vs Exiqon one color | 0.656 | 0.691 | -0.035 | 0.2848 |
| Exiqon two color vs LC Sciences | 0.656 | 0.506 | 0.151 | 0.0594 |
| Exiqon two color vs NCode one color | 0.656 | 0.730 | -0.073 | 0.3644 |
| Exiqon two color vs NCode two color | 0.656 | 0.775 | -0.119 | 0.1296 |
| NCode one color vs LC Sciences | 0.730 | 0.506 | 0.224 | 0.0338 |
| NCode two color vs LC Sciences | 0.775 | 0.506 | 0.270 | 0.0244 |
| NCode two color vs NCode one color | 0.775 | 0.730 | 0.046 | 0.2530 |
